# Improving and correcting the contiguity of long-read genome assemblies of three plant species using optical mapping and chromosome conformation capture data

**DOI:** 10.1101/gr.213652.116

**Published:** 2017-05

**Authors:** Wen-Biao Jiao, Gonzalo Garcia Accinelli, Benjamin Hartwig, Christiane Kiefer, David Baker, Edouard Severing, Eva-Maria Willing, Mathieu Piednoel, Stefan Woetzel, Eva Madrid-Herrero, Bruno Huettel, Ulrike Hümann, Richard Reinhard, Marcus A. Koch, Daniel Swan, Bernardo Clavijo, George Coupland, Korbinian Schneeberger

**Affiliations:** 1 Department of Plant Developmental Biology, Max Planck Institute for Plant Breeding Research, 50829 Cologne, Germany;; 2 Earlham Institute, Norwich Research Park, Norwich NR4 7UH, United Kingdom;; 3 Max Planck-Genome-center Cologne, 50829 Cologne, Germany;; 4 Department of Biodiversity and Plant Systematics, Centre for Organismal Studies (COS) Heidelberg, Heidelberg University, 69120 Heidelberg, Germany

## Abstract

Long-read sequencing can overcome the weaknesses of short reads in the assembly of eukaryotic genomes; however, at present additional scaffolding is needed to achieve chromosome-level assemblies. We generated Pacific Biosciences (PacBio) long-read data of the genomes of three relatives of the model plant *Arabidopsis thaliana* and assembled all three genomes into only a few hundred contigs. To improve the contiguities of these assemblies, we generated BioNano Genomics optical mapping and Dovetail Genomics chromosome conformation capture data for genome scaffolding. Despite their technical differences, optical mapping and chromosome conformation capture performed similarly and doubled N50 values. After improving both integration methods, assembly contiguity reached chromosome-arm-levels. We rigorously assessed the quality of contigs and scaffolds using Illumina mate-pair libraries and genetic map information. This showed that PacBio assemblies have high sequence accuracy but can contain several misassemblies, which join unlinked regions of the genome. Most, but not all, of these misjoints were removed during the integration of the optical mapping and chromosome conformation capture data. Even though none of the centromeres were fully assembled, the scaffolds revealed large parts of some centromeric regions, even including some of the heterochromatic regions, which are not present in gold standard reference sequences.

Contiguity of genome assemblies does not only ensure completeness of assemblies but is also essential to any kind of linkage or structural variation analysis. Short reads, up to a few hundred base pairs in length, are typically not sufficient to assemble eukaryotic sequences at high contiguities. Currently two different technologies can generate long-read sequence data from single molecules at sufficient throughput. The sequencing technologies of Pacific Biosciences (PacBio) (Eid et al. 2009) and Oxford Nanopore produce reads of up to 20 kb on average, and though the reads of these technologies have high error rates of up to 15%, the accuracy of assembled sequences can be as accurate as the early gold standard reference genome sequences (Quick et al. 2014; Berlin et al. 2015; Koren and Phillippy 2015).

Long-read sequencing can overcome the limitations of short reads by spanning many of the repetitive regions, which are presumably the main reason for the numerous breaks in short-read assemblies. In particular, the assembly of plant genomes, which have high levels of repetitive transposable elements and have significantly more repetitive *k*-mers compared to mammalian genomes (Nordström et al. 2013), is challenging with short reads. Recently, the first assemblies of plant genomes exclusively based on PacBio sequences were published, including assemblies of *Arabidopsis thaliana* (Landsberg *erecta*) with 38 contigs and an N50 of 11.2 Mb (Berlin et al. 2015) and of *Oropetium thomaeum*, a grass species having the smallest known grass genome of ∼250 Mb, with 625 contigs and an N50 of 2.4 Mb (VanBuren et al. 2015).

However, in order to arrange these contigs in their chromosomal context, mapping information that links the contigs to their original locations is required. Traditionally, genetic maps were used, but as significant parts of genomes can be heterochromatic and do not undergo meiotic recombination, contigs from such regions remain unordered. In addition, the initial generation of genetic maps is time-consuming and tedious. Alternatively, cytogenetic methods, such as fluorescence in situ hybridization (FISH) and comparative genomic hybridization can link contigs to their approximate genomic regions (Schranz et al. 2006), which, however, is also labor-intensive and does not come at high resolution (Willing et al. 2015).

An alternative way to arrange contigs is to generate read pairs sequenced from the two ends of a molecule of approximately known size, which can be used to order and orientate contigs (Roach et al. 1995). Though this does not map the resulting scaffolds to their chromosomal regions, sequencing the ends of medium-sized or long molecules, such as BAC ends, can immensely help to increase the contiguity of sequence assemblies.

Two recently introduced methods greatly improve the generation of such scaffolding data and promise reconstruction of entire chromosomes. The first technology, optical mapping, was already invented at the end of the last century (Schwartz et al. 1993), but recent automation of this process has led to the development of commercial high-throughput platforms, such as the Irys system released by BioNano Genomics (Tang et al. 2015). In general, optical mapping generates fingerprints of DNA sequences of several hundred kb in size by imaging the locations of the restriction sites under light microscopes using fluorescent labels (Lam et al. 2012). Such individual fingerprints can be further assembled to construct genome-wide maps, which can then guide the order and orientation of sequence contigs. The second technology, introduced in 2015 by Dovetail Genomics, is called the *Chicago* approach (Putnam et al. 2016). This method is based on the Hi-C technology (sequencing of read pairs generated by proximity ligation of DNA in natural chromatin), but simplified, using in vitro reconstituted chromatin. Such data produces links between genomic regions that can be up to several hundred kb apart and thus are useful for long-range scaffolding. After integration into a sequence assembly, it has been shown to generate N50 values that can be as large as 30 Mb (Putnam et al. 2016).

Though it is common to compare contiguity statistics (like the N50 value) of different assemblies, there are hardly any direct comparisons of the performance of different technologies on the same genomes, or comparison of the same technology (including identical application of it) on multiple genomes with different characteristics. Here, we present PacBio assemblies and the integration of BioNano Genomics’ optical mapping data of three relatives of the plant model *A. thaliana*. For one of the genomes, we have acquired additional Dovetail Genomics’ chromosome conformation capture data. The three genomes have drastic differences in genome size and the amount of repetitive sequence. This allowed us to compare the assembly performance of long-read data with and without optical mapping and chromosome conformation capture data in different scenarios and to develop general improvements for the integration of such long-range scaffolding data. The contiguities of contigs and scaffolds were carefully controlled before and after integration of the scaffolding information using short-read alignments, Illumina mate-pair libraries with different insert sizes, and a high-density genetic map. Integration of optical mapping and chromosome conformation capture data resolved most of the assembly errors, which were apparent in the initial PacBio assemblies and increased assembly contiguity to unprecedented levels, including scaffolds which spanned entire chromosome arms.

## Results

### Long-read assembly of three plant genomes

We have generated PacBio sequencing data for three diploid, inbred genomes of the Brassicaceae plant family (*Arabis alpina*, *Euclidium syriacum*, and *Conringia planisiliqua*). The two latter species have been selected because they represent different evolutionary lineages of the family, complementing already well-established species such as *A. thaliana*, including Lineage III from which no species has been assembled so far, whereas *A. alpina* is an emerging model for perennial flowering studies. The read data were generated with P6-C4 chemistry on a PacBio RS II machine with an average filtered subread length of 8.5, 6.9, and 7.9 kb (Supplemental Fig. S1; Supplemental Table S1). Based on estimated genome sizes of 370, 262, and 224 Mb, respectively (Hohmann et al. 2015), sequence coverage was around 86×, 47×, and 54× for these three species.

**Figure 1.**
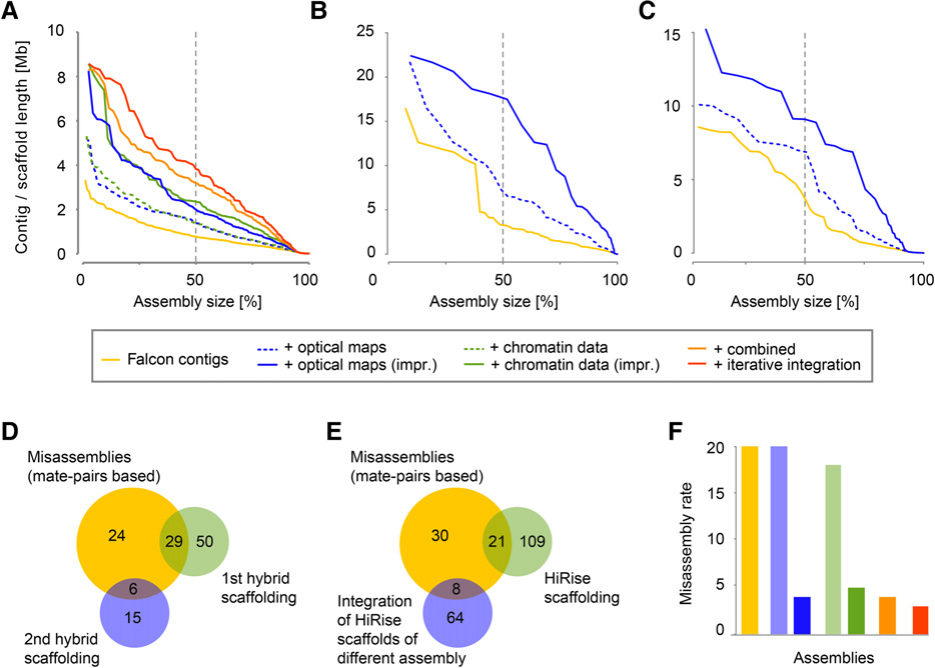
Assembly results and strategies. (*A*–*C*) Assembly contiguity of the assemblies of three species: *A. alpina* (*A*), *E. syriacum* (*B*), *C. planisiliqua* (*C*). The *x*-axis indicates the cumulative length of contigs sorted by length (expressed as percent of the entire assembly). The *y*-axis shows individual contig or scaffold length. The dashed line indicates the N50/L50 values. (*D*) Misassemblies identified with Illumina mate-pairs (yellow) and their overlap with breaks introduced during misassembly identification using optical maps (in two steps shown in green and blue). (*E*) Misassemblies identified with Illumina mate-pair alignments (yellow) and their overlap with breaks introduced during our integration of Dovetail Genomics chromosome conformation capture data (again, two steps shown in green and blue). (*F*) Inter-chromosome misassemblies identified by a genetic map in each of the assemblies (as shown in *A*).

We used two different tools, FALCON (Chin et al. 2016) and PBcR (Berlin et al. 2015), for whole-genome assembly. Each of the six whole-genome assemblies, one for each combination of assembly tool and genome, was followed by two correction steps, one with long reads using Quiver (Chin et al. 2013) and one based on alignments of Illumina short reads (Table 1). Across all assemblies, FALCON assembled the data into fewer contigs as compared to PBcR, which was most drastic for *E. syriacum*, where FALCON generated only around one-fourth of the contigs generated by PBcR. The total lengths of the assembled sequence, however, were very similar between the assemblies, with the exception of *A. alpina*, where PBcR assembled 19 Mb more sequence. FALCON generated N50 values of 770 kb, 3.3, and 3.6 Mb for *A. alpina*, *E. syriacum*, and *C. planisiliqua* (L50: 121, 14 and 14) respectively, as compared to the PBcR assemblies with N50 values of 914 kb, 975 kb, and 1.5 Mb (L50: 99, 51 and 23) (Fig. 1A–C). Accordingly, contiguity of the assemblies was negatively correlated to genome size and not to sequence coverage, suggesting that genome complexity rather than amount of sequence data was limiting the assembly performance.

**Table 1.**
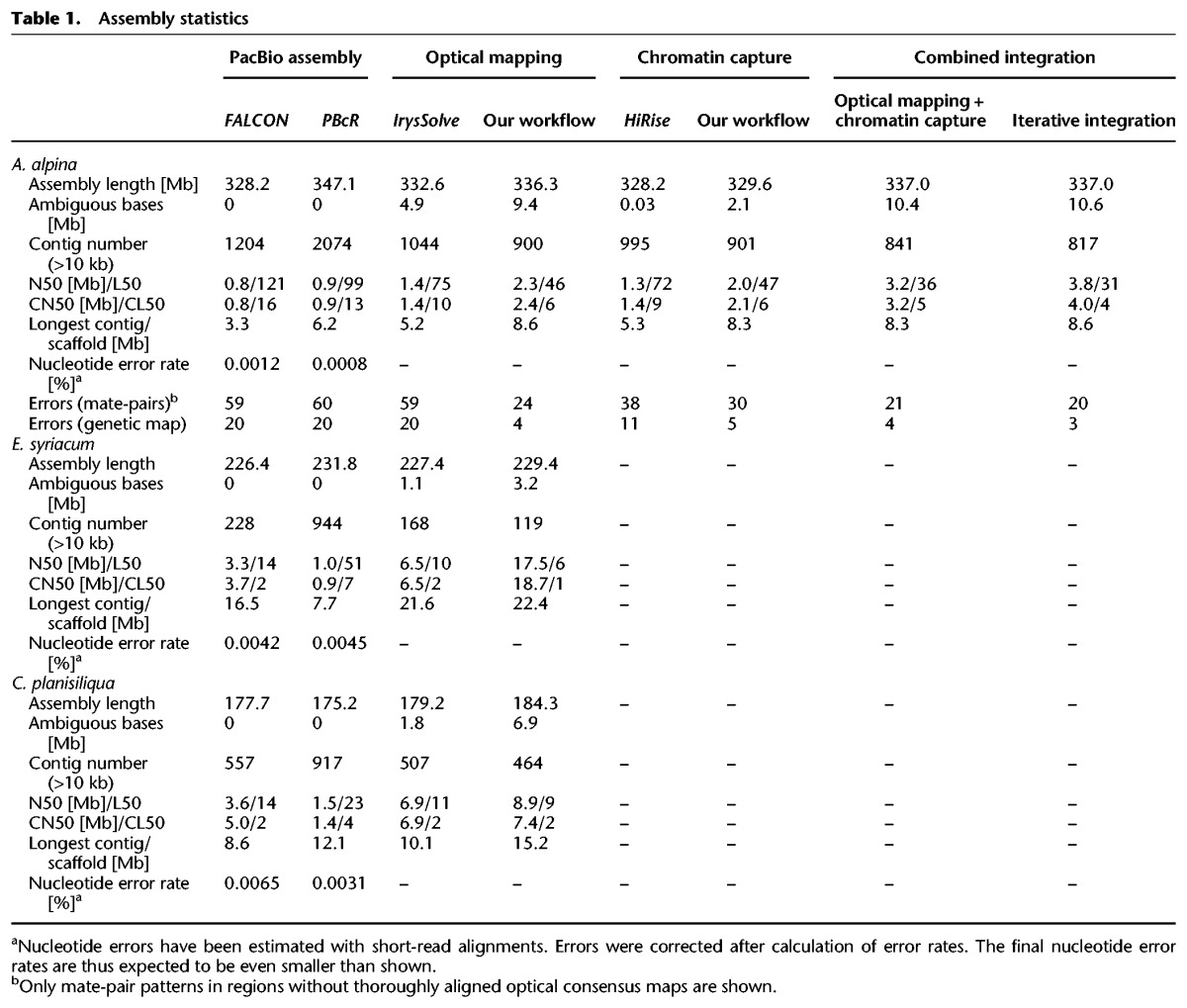
Assembly statistics

In general, assembly statistics of genomes with multiple chromosomes cannot reach their theoretical optimum (for example, in the case of L50, this would be 1) as even in a perfect assembly the finished genome sequence would be characterized by multiple contigs representing the individual chromosomes. This effect is marginal if an assembly consists of many contigs; however, if the contig number is low, this affects the interpretation of L50 values. To overcome this, we introduced *chromosome-N50* (CN50) and *chromosome-L50* (CL50), which estimate the median assembly contiguity (N50) of each chromosome, assuming chromosomes of equal length and assembly quality. Interestingly, the length of the CN50 contig is typically similar to the length of the N50 contig; however, its order number (CL50) will be with respect to a chromosome and thus can reach its optimum of 1 independent of chromosome number. For example, the L50 of the FALCON assembly of *C. planisiliqua* was 14, whereas the CL50 value was 2. This CL50 value illustrates that half of the average chromosome was assembled into not more than two contigs, a fact that is not apparent from the L50 value alone. See Methods and Supplemental Figure S2 for details.

### Assembly quality and contiguity control

We estimated the single-nucleotide error rates in the assemblies using the Illumina short-read alignments performed for genome polishing but estimated the error before the actual genome polishing. Even though this implies that the actual error rates are even smaller than estimated, the estimated error rates were already extremely low across all six assemblies (Table 1). Most of the errors found in both assemblies were small indels, which probably arose from the raw sequencing reads as indels are the most common type of sequencing error in PacBio reads (Supplemental Table S2) and as heterozygosity, another factor potentially introducing errors, was generally very low (*A. alpina*: 0.086%; *C. planisiliqua*: 0.061%; *E. syriacum*: 0.045%).

**Figure 2.**
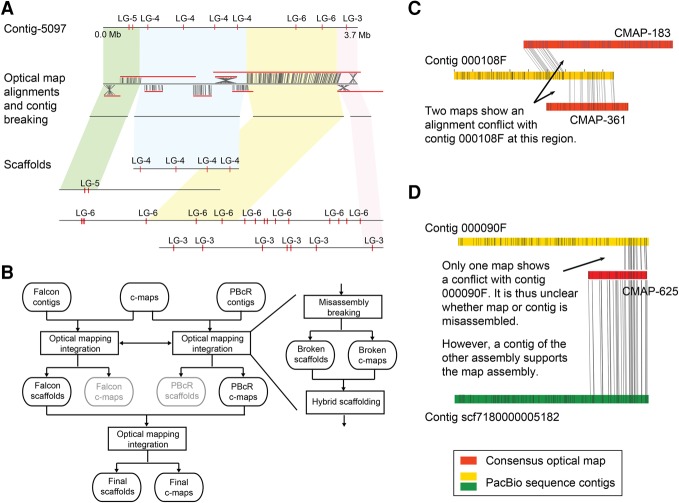
Optical mapping-based assembly correction and scaffolding. (*A*) Example of misassembly breakage and new scaffolding using optical mapping data. Three misassemblies in contig-5097 were identified with the optical map alignments (and also validated by the genetic maps; markers shown with red ticks). The original contig was broken, and the subsequent scaffolding of the four contigs, which resulted from breaking the original contig at the misassemblies, introduced them into the context of larger scaffolds, which were supported by the genetic map. (LG) Linkage group. (*B*) Improved optical mapping scaffolding workflow. Integration of optical mapping information includes breakage of misassembled contigs and consensus maps (c-maps) followed by hybrid scaffolding. (*C*) FALCON contig 000108F is apparently misassembled, as two different consensus maps (CMAP-183 and CMAP-361) have conflicting alignments with the same region of this contig. (*D*) A conflict between FALCON contig 000090F and CMAP-625 is not sufficient to decide on the origin of the underlying misassembly. However, CMAP-625 can be fully aligned to contig scf7180000005182 of a different (PBcR) assembly, supporting the correctness of this consensus map and thereby suggesting a misassembly in the contig.

However, not all misassemblies lead to single-nucleotide errors; some wrongly assembled regions might also join unlinked regions (e.g., through shared repeats) and thereby introduce severe artifacts to the assembly (example shown in Fig. 2A). To find those, we generated three Illumina mate-pair libraries with average insert sizes of 5, 7, and 10 kb for *A. alpina* (Supplemental Fig. S3), and we screened for mate-pairs where the two reads were aligned to two different contigs, including at least one of the reads being aligned to the inner part of the contig (Supplemental Table S3). Across all three libraries, we found 59 such regions in each of the FALCON and PBcR assemblies of *A. alpina* (Fig. 1D,E). As wrong alignments of the mate-pairs could potentially introduce false patterns and thereby artificially increase the number of misassemblies, we additionally generated a genetic map from 389 *A. alpina* F_2_ individuals derived from a cross of two diverse accessions. We aligned the sequences of 734 markers to the contigs of both *A. alpina* assemblies and screened for contigs with markers from different linkage groups to find inter-chromosomal misassemblies (Supplemental Table S4). In both the FALCON and the PBcR assemblies of *A. alpina*, we found 20 such misassemblies (on 19 and on 15 contigs, respectively), which were not shared between each other (Fig. 1F).

### Optical mapping data integration

For each of the three species, we generated optical mapping data using BioNano Genomics technology. Overall, we mapped 1.7, 0.8, and 0.5 million single molecules with an average length of 157, 145, and 200 kb, representing 722, 446, and 410× genome coverage for *A. alpina*, *E. syriacum,* and *C. planisiliqua*, respectively (Supplemental Table S5; Supplemental Fig. S4). Single-molecule maps were assembled into consensus maps using BioNano Genomics’ IrysSolve software with N50 values from 625 kb to 1.5 Mb.

We first aligned the consensus maps to the FALCON contigs (Supplemental Table S6). Overall, most of the consensus maps could be reliably aligned; however, the alignments also revealed 79, 10, and 23 conflicts, respectively, with the sequence contigs of *A. alpina*, *E. syriacum*, and *C. planisiliqua*. Similarly, alignments against the PBcR contigs revealed 69, 41, and 25 conflicts.

Following the standard workflow of BioNano's Irys software, we removed the alignments of all conflicting consensus maps before using the remaining alignments for hybrid scaffolding. This merged 253, 80, and 67 FALCON contigs and improved CN50 values of the three assemblies to 1.4, 6.5, and 6.9 Mb (Table 1). However, only removing the alignments of the conflicting consensus maps keeps the conflicting contigs in the assemblies and does not resolve putative misassemblies.

To improve this, we first assessed for each conflict if a misassembly of the consensus map or of the contig was the reason for the conflict (Fig. 2). For this, we first checked if the conflicting contig showed additional conflicts with other consensus maps, which could indicate an misassembly of the contig (Fig. 2C). If this was not the case, we checked if a contig of the assembly generated with the other assembly tool matched the conflicting consensus map, which again would indicate that the contig and not the consensus map was misassembled (Fig. 2D). In the opposite case, if the contig of the other assembly would reveal the same conflict, a misassembly of the consensus map would have been revealed. In any other case, we did not assign the assembly error to either the maps or contigs but flagged both as potentially misassembled. Across all three FALCON assemblies, in 93% of the conflicts that could be assigned to originate either from a sequence or map misassembly, it was the sequence that was wrongly assembled, and only in around 7% of the conflicts the consensus map assembly was wrong. The misassembled regions were significantly enriched for transposable elements, suggesting that the predominant reason for misassemblies is, in fact, repeats which were not resolved accurately during sequence assembly (Supplemental Fig. S5; Supplemental Table S7).

Instead of removing the alignments of these contigs and maps, we broke them at the misassembled regions. Subsequent hybrid scaffolding generated assemblies with CN50 values of 1.6, 8.9, and 7.4 Mb for the three species. We repeated this for the PBcR assemblies with similar outcomes. Interestingly, breaking the *A. alpina* FALCON contigs removed 19 (95%) misassemblies that we had identified with the genetic map and 29 (49%) of the misjoints that were revealed with the mate-pair alignments.

To improve this integration even further, we used the hybrid consensus maps that were generated by integrating the optical mapping data into the PBcR contigs for a second round of hybrid scaffolding of the FALCON-based scaffolds (Fig. 2B). Though this second hybrid scaffolding is based on the identical optical mapping data, the consensus maps generated during hybrid scaffolding of the PBcR contigs included connections which have not been introduced into the FALCON scaffolds. This final integration further improved the CN50 to 2.4, 18.7, and 7.4 Mb (Table 1; Fig. 1A–C) and removed 19 (95%) and 35 (59%) of the misassemblies found with the genetic map and the mate-pairs. This implied that our workflow did not only increase assembly contiguity, but also substantially improved assembly quality, even though we also found three additional misassemblies in the scaffolds of *A. alpina* that were introduced during the second round of hybrid scaffolding. The CL50 values of the assemblies were 6, 1, and 2, indicating that some of the chromosome arms were fully assembled.

### Chromosome conformation capture data integration

For *A. alpina*, we also ordered chromosome conformation capture data from Dovetail Genomics. This service provider offers DNA extraction, library preparation, and sequencing of read pairs generated from proximity ligation of DNA from in vitro reconstituted chromatin (Putnam et al. 2016). Read pairs which are close in in vitro chromatin are also physically close and thus can be used for assembly scaffolding.

Overall, 155.8 million read pairs were generated, and around 39% and 40% of these read pairs could be aligned to the initial FALCON and PBcR contigs, including 8.4% and 9.0% that aligned to different contigs. The distance distribution of read pairs reached up to multiple hundred kb, including 1.3% of the read pairs with a distance larger than 25 kb (Supplemental Fig. S6).

We first performed genome scaffolding using Dovetail Genomics’ HiRise software. During integration of the read-pair information, putatively misassembled regions are first identified and the underlying contigs are broken. The error-corrected contigs are then scaffolded using read pairs aligned to different contigs. HiRise scaffolding improved CN50 from 771 kb to 1.4 Mb for the *A. alpina* FALCON assembly (Table 1). Using the genetic map again, we found that only four of the 20 misassemblies were removed and that two additional misassemblies were introduced, leading to 18 misassemblies in total. The results were similar for the misassemblies identified with the mate-pair data—21 out of 59 misassemblies were identified, broken, and removed.

**Figure 3.**
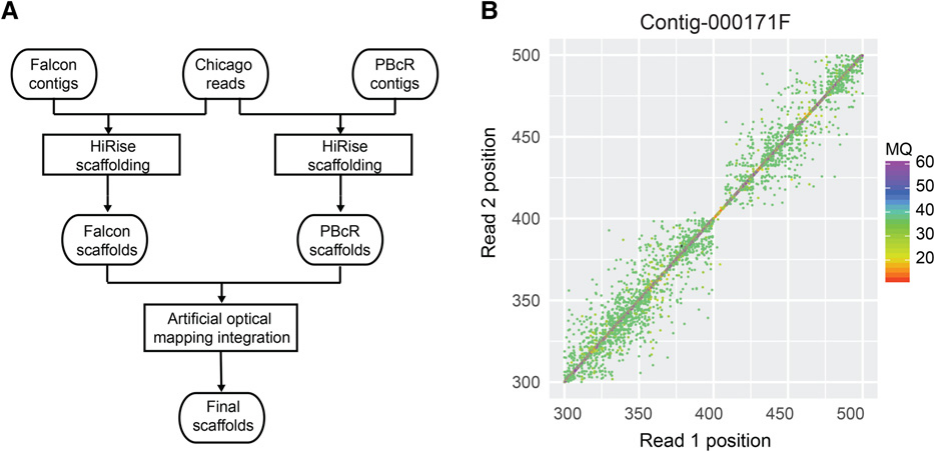
Assembly scaffolding using chromosome conformation capture data. (*A*) Improved chromosome conformation capture data scaffolding workflow. (*B*) Misassembly identification using chromosome conformation capture read pairs. The paired-end mapping positions in the region 300–500 kb of FALCON contig 000171F show a sudden absence of read pairs spanning across the region at around 410 kb. A misassembly at this region was indicated by HiRise. (MQ) Mapping quality.

As the earlier integration of the optical mapping data was improved by combining hybrid maps from different assembly integrations, we tried to advance chromosome conformation capture data-based scaffolding by again combining the improvements of two independent scaffoldings. For this, we transformed the HiRise PBcR scaffolds into artificial in silico optical maps (Fig. 3). This allowed us to integrate the HiRise PBcR scaffolds into the HiRise FALCON scaffolds following our hybrid scaffolding method introduced for optical mapping data. This increased contiguity of the scaffolds to a CN50 value of 2.1 Mb (Fig. 1A). Moreover, this improved integration of the chromosome conformation capture data removed 19 of the 20 misassemblies found with the genetic map and 29 of the 59 misassembled regions found with the mate-pairs (Fig. 1E). However, similarly to the integration of the optical mapping data, we found four additional misassemblies that were introduced during our improved way of scaffolding.

### Comparing and combining optical mapping and chromosome conformation capture data

As described, we performed independent integrations of both BioNano Genomics’ optical mapping and Dovetail Genomics’ scaffolding data into two PacBio assemblies of *A. alpina* following the standard procedures as well as improved ways of integration. The contiguity of the two different assemblies after the initial integrations was surprisingly similar, and even after introducing modifications to the integration methods, the contiguity of both assemblies remained very similar (Fig. 1A).

However, similar scaffolding performance of optical mapping and chromosome conformation capture data does not necessarily imply that their scaffolding information was redundant. As these technologies suffer from different challenges, they promise to improve scaffolding even further if integrated together. For example, typical breakpoints in chromatin capture maps are long tandem repeat arrays, which can be spanned by optical maps. In contrast, closely linked restriction sites can introduce double-strand breaks in DNA prepared for optical mapping, not affecting the chromosome conformation capture data (Pendleton et al. 2015).

We therefore integrated the chromosome conformation capture data into the most contiguous optical mapping-based scaffolds of *A. alpina* using HiRise. This increased CN50 from 2.4 to 3.2 Mb and decreased CL50 from 6 to 5, corroborating that the contiguity information provided by both technologies was not fully redundant (Table 1).

As HiRise was very conservative regarding breaking contigs at putative misassemblies, we reran an additional integration of the hybrid consensus map to merge falsely broken contigs during HiRise integration and to assemble the most contiguous scaffolds. This final assembly of *A. alpina*, which was based on iterative integration of the scaffolding data, had unprecedented CN50 and CL50 values for this assembly of 4.0 Mb and 4 and a maximum contig length of 8.6 Mb (Fig. 1A; Table 1), implying that half of each chromosome was assembled into four scaffolds only. The error rate was also further reduced; all 20 errors that were originally included in the FALCON contigs were resolved, while only three new misassemblies were introduced during scaffolding. Likewise, 39 (66%) of all regions, where mate-pair alignments indicated putative misassemblies in the contigs, were broken in this final assembly.

### Assembly of chromosomes

Assembly of entire chromosomes requires assembly of centromeric regions. Centromeric regions are usually highly repetitive as they are hotspots for transposable element insertion and often they include centromeric repeats, which are tandem repeat arrays of short sequences of up to hundreds of kb in size (Henikoff et al. 2001).

To analyze what parts of the chromosomes are captured in the scaffolds, we first screened for highly abundant tandem repeats, as these typically include candidates for the centromeric repeats (Melters et al. 2013). Secondly, we used whole-genome comparisons against *Arabidopsis lyrata* (Hu et al. 2011) to identify regions with homology to centromeric regions. The eight chromosomes of *A. lyrata* represent the ancestral karyotype of the Brassicaceae family (Schranz et al. 2006) and usually share at least some conserved centromeres with other Brassicaceae species. Thirdly, we analyzed the repeat and gene densities across each scaffold, as a typical Brassicaceae chromosome has low repeat and high gene density at its euchromatic ends and high repeat and low gene density around the centromere.

The most abundant tandem repeats within each of the three assemblies included, besides centromeric repeat candidates, arrays of rDNA repeats (Supplemental Table S8). Almost of all these rDNA repeats were found on short scaffolds with sequence similarity to rDNA across nearly their complete length. The others could be found at the end of contigs, implying that none of these arrays was fully assembled. This suggested that tandem repeats are, in addition to transposable elements, another reason for assembly breakage.

We found clear candidates for centromeric repeats for *A. alpina* and *C. planisiliqua*; *E. syriacum*, however, lacked any obvious candidates as all non-rDNA tandem repeats were of low abundance (Supplemental Table S8). The centromeric repeat monomers for *A. alpina* and *C. planisiliqua* were 496 and 221 bp long and occurred with higher order in some repeat arrays (Melters et al. 2013). Most of the scaffolds that included centromeric repeat arrays included multiple closely linked arrays. These clusters of centromeric repeat arrays were usually close to the end of scaffolds or spanned entire scaffolds, as in the most extreme case, where one 690-kb scaffold of *A. alpina* harbored 23 centromeric repeat arrays across its entire span. This again shows that tandem repeats are hard to assemble; however, some of such centromeric repeat clusters in the *C. planisiliqua* scaffolds resided in the middle of scaffolds, suggesting the assembly of major parts of some centromeric regions.

**Figure 4.**
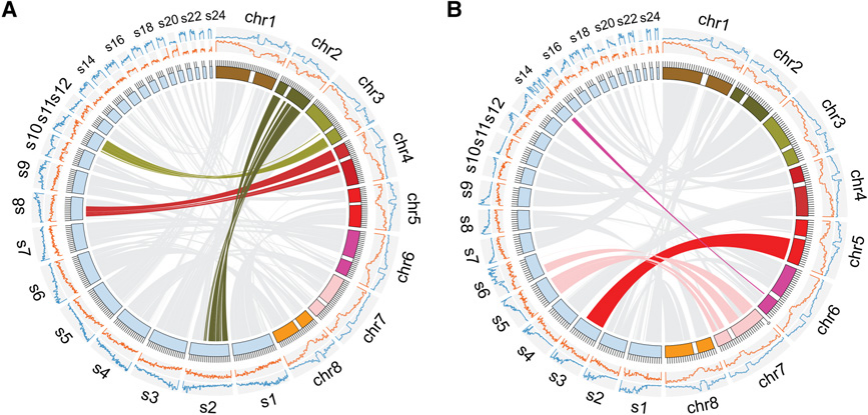
Comparing the assemblies of *E. syriacum* and *C. planisiliqua* to the ancestral karyotype present in the genome of *A. lyrata.* The eight chromosomes of *A. lyrata* are shown in colored blocks. Centromeric regions are indicated by white breaks. Scaffolds of the assemblies generated here of more than 1 Mb are shown in light blue blocks. The two histograms outside of chromosome karyotypes show the gene (orange) and repeat (blue) densities assessed with window sizes of 1 Mb for *A. lyrata* and 200 kb for *E. syriacum* or *C. planisiliqua*. (*A*) Three scaffolds of *E. syriacum* include similarities to the two flanking regions of *A. lyrata* CEN2, CEN3, and CEN4. (*B*) Scaffolds 3, 5, 6, and 14 include up to 7 Mb of putative centromeric regions, which are absent in the core assembly of *A. lyrata*, as these regions do not show any homology to any region in the *A. lyrata* genome.

Even though the absence of centromeric repeats in the *E. syriacum* assembly did not support the assembly of any centromeric regions, we found that scaffold-2 showed homology across the entire *A. lyrata* Chromosome 2 and even large parts of Chromosome 1 (Fig. 4A)*.* However, scaffold-2 also showed a steady increase of the repeat density from one end to the other and gene density that increased in the opposite direction, which did not resemble the usual chromosome structure. This suggested that, even though the scaffold assembled through an ancestral chromosome, it only represents a chromosome arm and that chromosomal rearrangements during the evolution of *E. syriacum* removed this ancestral CEN 2. This was further supported by the fact that only the gene-rich end of the scaffold featured telomeric repeats (Supplemental Table S9). In addition, there were two scaffolds with homology to complete ancestral centromeres (CEN3 and CEN4), but also in these cases, it seems more likely that the scaffolds do not represent entire chromosomes, as again the gene and repeat densities did not resemble the common chromosome structures and also lacked telomeric repeats at both respective ends.

For *A. alpina* and *C. planisiliqua*, we could not find any scaffold with homology across entire ancestral centromeres. However, the assembly of *C. planisiliqua* included four large regions of up to 7 Mb of sequence (on scaffold-3, -5, -6, and -14) which were not homologous to any region in the *A. lyrata* genome. All four sequences were located in regions in which the centromeres were estimated, suggesting that the assembly of *C. planisiliqua* included parts of the centromeric sequence that were not even included in the assembly of *A. lyrata* (Fig. 4B). In each of the four regions, we found centromeric repeat arrays, further supporting that these regions represent partially assembled centromeres.

### Assembly finalization and gene annotations

The scaffolds of the *A. alpina* assembly were further arranged into eight pseudo-molecules using genetic and cytogenetic maps, following the same steps as in Willing et al. (2015). In addition, we performed gene and transposable element annotations for each species to increase usability of the final assemblies (Supplemental Tables S10, S11). To check which assembly steps were impacting most on the quality of the gene annotations, we searched each of the intermediate assemblies for genes (Supplemental Tables S12, S13). Already, after assembly polishing using the PacBio reads, more them 97% of the genes in *E. syriacum* and *C. planisiliqua* could be identified. In *A. alpina*, where PacBio read depth was higher, even 99.5% of the genes were present. Most of the remaining gene sequences were then established by the assembly corrections with short reads, and only 0.003%–0.17% of genes were not present after Illumina correction. However, within the raw PacBio assemblies, 54%–64% of the gene sequences could not be found, underlining again the importance of assembly corrections.

## Discussion

We presented the first PacBio-only genome assemblies of three relatives of *Arabidopsis* in the Brassicaceae family. Further integration of optical mapping and chromosome conformation capture data generated scaffolds that reconstructed entire chromosome arms. To compare assemblies that start to reach maximal contiguity, we have introduced a new assembly statistic called chromosome-N50 (CN50) to express the assembly quality on a chromosomal level.

The nucleotide error rates within our assemblies were lower than 1 in 10 kb, which is similar to the accuracy of the Sanger sequencing-based reference sequences released nearly 20 years ago. The most severe misassemblies, however, do not result from accumulation of per-base errors but from connections of unlinked regions. We identified such misassemblies in the PacBio contigs; however, integration of optical mapping and chromosome conformation capture data helped to remove most of them. In our analysis, the majority of conflicts between sequence contigs and optical consensus maps were due to errors in the sequence assembly and not due to errors in the optical map assemblies. It might be that a more rigorous assembly of the optical maps would lead to more error-prone optical consensus maps. However, as these additional errors could be corrected during integration into the sequence assembly, generation of rigorous optical map assemblies might be one way to further improve the assembly contiguity of the final sequence assembly without adding many misassemblies.

Integration of optical map and chromosome conformation capture data was complementary, and combined usage led to an assembly that was better than using either technology in isolation. The integration of optical maps relies on contigs of sufficient minimal lengths. Short contigs often have too few nick sites and cannot be reliably aligned to optical maps, whereas scaffolding using chromatin contact data is less affected by contig size, and, in fact, it was possible to scaffold more of the short contigs using chromosome conformation data as compared to optical maps.

Assembly contiguity was improved not only by scaffolding with the optical mapping or chromosome conformation capture data alone but also by integrating the contiguity of assemblies generated with a different assembly tool. This implied that both tools, FALCON and PBcR, assembled regions that were not assembled by the respective other tool, suggesting that both assembly algorithms could still be improved and that the data contains even more overlap information than what we can obtain by using current tools.

It needs to be determined if further advancements in long-read sequencing will make scaffolding obsolete in the near future or if long-range technologies will be commonly used to supplement sequence assembly. Future genome assembly might still profit from improved methods for integration of sequence and scaffolding data, including inclusion of all information into initial assembly graphs before untangling them into individual contigs. Using the scaffolding information during this untangling process would allow the performance of assembly and scaffolding as a one-step procedure and with this, improve sequence assembly as well as the scaffolding of the contigs.

## Methods

### Plant selection

Besides their phylogenetic origins, plant species have been selected following various criteria: (1) diploid species with (2) significant different genomes, and (3) self-compatible accessions. *A. alpina* is a diploid (2*n* = 16), often selfing species from tribe Arabideae (expanded evolutionary lineage II). *E. syriacum* is a diploid and selfing species (2*n* = 14) from tribe Euclidieae (evolutionary lineage III), and *C. planisiliqua* is a selfing, diploid species (2*n* = 14) from tribe Conringieae (expanded evolutionary lineage II). *C. planisiliqua* seed material was obtained from the *BrassiBase* database (http:// brassibase.cos.uni-heidelberg.de; Koch et al. 2012; Kiefer et al. 2014) and is fully documented with seed accession code HEID921022 (herbarium voucher HEID503985). *E. syriacum* seed material was obtained from the Kew Millenium Seedbank database with accession code KEW653912. For *A. alpina*, offspring of the reference accession Pajares were used (Willing et al. 2015). For detailed sample preparation, see Supplemental Methods.

### PacBio assembly

All PacBio sequencing was performed on a PacBio RS II sequencer using P6C4 sequencing reagents. For more details, see Supplemental Methods. The PacBio reads for *A. alpina*, *C. planisiliqua*, and *E. syriacum* were imported into the SMRT Analysis software (v2.3) to remove subreads shorter than 500 bp or with a quality (QV) <80. Filtered subreads were then used for de novo assembly with FALCON (v0.3.0) and PBcR (with Celera Assembler 8.3rc2). For FALCON, we set minimal read-length values in the read correction and assembly steps to keep the combined lengths of the input reads close to 25× as recommended. For PBcR, we selected a subset of the filtered subreads with a combined genome coverage of 40× for read correction with MHAP and 25× of the longest, corrected subreads for the overlap-layout-consensus assembly with the Celera Assembler. Assembled contigs from FALCON and PBcR were polished by mapping the filtered subreads, followed by a consensus analysis using Quiver.

### Assembly error-rate estimations

For assembly error-rate estimations, we generated sequencing data from Illumina paired-end and mate-pair libraries. A genetic map of *A. alpina* was generated to estimate inter-chromosome misassemblies. See Supplemental Methods for details on data generation. We estimated assembly accuracy at the nucleotide level using Illumina paired-end short-read alignments generated with BWA (v 0.7.12) (Li and Durbin 2009), and SNPs and indels were called with SAMtools (Li et al. 2009). Assembly error rates were estimated by dividing the number of homozygous SNPs and indels by the total length of covered regions with mapping quality of more than 25 and a coverage of more than five.

The level of more complex errors was estimated with Illumina mate-pair libraries. First, we mapped the reads to each of the assemblies using BWA and calculated the insert size distribution for each of the three libraries. Then, we clustered read pairs wherein the two reads of each pair were aligned to different contigs but where the reads of each of the pairs were aligned to the same two contigs with a distance of less than three standard deviations of the insert size distribution of the respective library. To decrease the effect of misalignments, we only used read alignments with mapping quality more than 30 and without any mismatches or indels. Each cluster of read pairs with more than five read pairs where at least one of the read clusters was aligned to the inner part of a contig revealed a misassembled region. Finally, the results of all three libraries were merged to remove redundant regions.

### Definition of CN50 and CL50

Let *C* be a length-sorted list of all contigs (from longest to shortest). Select *n* distinct sets of contigs, where *n* equals the number of chromosomes, such that the first (longest) contig is assigned to set 1, the second contig to set 2, and so on. The *n* + 1 longest contig is then assigned to set *n* again, and the *n* + 2 longest contig is assigned to set *n* − 1, and so on. Let *S* describe a length-sorted list of contigs of set *s*. For each set, select one contig *c*_*i*_ ∈ *S* such that

$$\overset{i}{\underset{k=1}{\sum }}\text{length}(c_{k})\geq \frac{\sum_{k=1}^{\left|S\right|}\text{length}(c_{k})}{2},$$

where *i* ∈ *S*, and no *j* < *i* exists, which fulfills the same criterion. Let *M* be the set of selected contigs. We define

$$CN50=\text{median}\left(\overset{\left|M\right|}{\underset{k=1}{⋃}}\text{length}(c_{k})\right),$$

where *c* ∈ *M*, and *CL50* is defined as the order number *i* of the CN50 contig *c*_*i*_ ∈ *S*.

### Optical map de novo assembly and hybrid scaffolding

Optical map data were generated by Earlham Institute's Platforms and Pipelines group. For details on data generation, see Supplemental Methods. For all three genomes, the optical consensus maps were de novo assembled with the Assembler tool of the IrysSolve package using significance cutoffs of *P* < 8 × 10^−8^ to generate draft consensus maps, *P* < 8 × 10^−9^ for draft consensus map extension, and *P* < 8 × 10^−12^ for final merging of the draft consensus maps. For hybrid scaffolding, we applied two different scaffolding strategies. For the first, we used RefAligner to align optical consensus maps to the assembly sequences with initial alignment cutoff of *P* < 1 × 10^−9^. Only consensus maps without conflicting alignments were utilized for hybrid scaffolding using the IrysSolve software. For the second integration, we included the consensus maps with conflicting alignments. However, we broke them (or the respective contigs) at the putatively misassembled regions, which were defined as the midpoint between the last aligned nick site and first unaligned nick site at the conflicting regions (and were further adjusted if an indel with at least two nick sites was close to this breakpoint). To decide whether the contig or the consensus maps were misassembled (and should be broken), we searched for conflicting alignments to the focal contigs or consensus map. If no additional conflicts were found in the first set of alignments, we extended this search to a more relaxed set of alignments using *P* < 5 × 10^−8^ as cutoff. If this still did not reveal the origin of the conflict, we finally checked the alignments of the consensus map to the contigs of the other assembly (FALCON or PBcR). If it was still not possible to determine whether the contig or the consensus map was misassembled, we split both contig and consensus map. Once all conflicts were resolved, we ran RefAligner (*P* < 1 × 10^−9^) again for hybrid scaffolding. This was performed for the FALCON as well as for the PBcR contig. As a last step of the second integration, we used RefAligner (*P* < 1 × 10^−9^) to align scaffolds from FALCON's hybrid scaffolding to the hybrid consensus maps from the PBcR hybrid scaffolding. We again broke misassembled scaffold sequences or maps as above, before generating the final hybrid scaffolding using alignments with *P* < 1 × 10^−9^.

For both scaffolding methods, we estimated the gap length between the contigs of a scaffold using the estimated length between the flanking restriction sites and filled the sequence with the respective number of Ns.

During revision of our manuscript, BioNano released an update of the Irys scaffolding software, which now includes misassembly correction based on the comparison of maps and contigs.

### Chromosome conformation capture read mapping and scaffolding

We aligned chromosome conformation capture reads to the *A. alpina* FALCON and PBcR assembly contigs using BWA (mapping quality cutoff of 30) and performed the assembly scaffolding using HiRise software after defining repetitive regions with Illumina short-read alignments. To improve the HiRise scaffolding results, we transformed the PBcR HiRise scaffolds into in silico consensus maps using the nick site of BspQI. These consensus maps were then aligned to FALCON scaffolds using RefAligner with *P* < 1 × 10^−9^ as a cutoff. To remove conflicting alignments, we then broke the FALCON scaffolds and PBcR in silico maps before scaffolding them as described above. As gap length is hard to determine due to the variable insert length of chromosome conformation capture read pairs, we introduced 100 Ns between each the contigs of each scaffold.

### Integration of optical mapping and chromosome conformation capture data

For integration of optical mapping and chromosome conformation capture data, we first followed our second optical mapping scaffolding workflow as described above, except that we also used the chromosome conformation capture data to decide whether the contig or the consensus map was misassembled in case of alignment conflicts. Secondly, we aligned chromosome conformation capture reads to the resulting hybrid scaffolds and performed HiRise scaffolding. After this, we again introduced the optical mapping information by aligning the hybrid consensus maps from the first step of optical mapping scaffolding to scaffolds from the second step of HiRise scaffolding (*P* < 1 × 10^−9^), followed by breaking of potentially misassembled maps and scaffolds, and final scaffolding.

### Scripts

Scripts to repeat all workflows introduced here can be found in the Supplemental Scripts and online (https://github.com/wen-biao/OM-HiC-scaffolding).

## Data access

Whole-genome sequencing, optical mapping, and chromosome conformation capture data as well as the final assemblies from this study have been submitted to the European Nucleotide Archive (ENA; http://www.ebi.ac.uk/ena) under the BioProject ID PRJEB16743. Gene annotations of *A. alpina* are available at www.arabis-alpina.org and also in the Supplemental Data, where annotations for all three genomes can be found.

## Supplementary Material

Supplemental Material

## References

[JIAOGR213652C1] Berlin K, Koren S, Chin C-S, Drake JP, Landolin JM, Phillippy AM. 2015 Assembling large genomes with single-molecule sequencing and locality-sensitive hashing. Nat Biotechnol 33: 623–630. 26006009 10.1038/nbt.3238

[JIAOGR213652C2] Chin C-S, Alexander DH, Marks P, Klammer AA, Drake J, Heiner C, Clum A, Copeland A, Huddleston J, Eichler EE, 2013 Nonhybrid, finished microbial genome assemblies from long-read SMRT sequencing data. Nat Methods 10: 563–569. 23644548 10.1038/nmeth.2474

[JIAOGR213652C3] Chin C-S, Peluso P, Sedlazeck FJ, Nattestad M, Concepcion GT, Clum A, Dunn C, O'Malley R, Figueroa-Balderas R, Morales-Cruz A, 2016 Phased diploid genome assembly with single-molecule real-time sequencing. Nat Methods 13: 1050–1054. 27749838 10.1038/nmeth.4035 PMC5503144

[JIAOGR213652C4] Eid J, Fehr A, Gray J, Luong K, Lyle J, Otto G, Peluso P, Rank D, Baybayan P, Bettman B, 2009 Real-time DNA sequencing from single polymerase molecules. Science 323: 133–138. 19023044 10.1126/science.1162986

[JIAOGR213652C5] Henikoff S, Ahmad K, Malik HS. 2001 The centromere paradox: stable inheritance with rapidly evolving DNA. Science 293: 1098–1102. 11498581 10.1126/science.1062939

[JIAOGR213652C6] Hohmann N, Wolf EM, Lysak MA, Koch MA. 2015 A time-calibrated road map of Brassicaceae species radiation and evolutionary history. Plant Cell 27: 2770–2784. 26410304 10.1105/tpc.15.00482 PMC4682323

[JIAOGR213652C7] Hu TT, Pattyn P, Bakker EG, Cao J, Cheng J-F, Clark RM, Fahlgren N, Fawcett JA, Grimwood J, Gundlach H, 2011 The *Arabidopsis lyrata* genome sequence and the basis of rapid genome size change. Nat Genet 43: 476–481. 21478890 10.1038/ng.807 PMC3083492

[JIAOGR213652C8] Kiefer M, Schmickl R, German DA, Mandáková T, Lysak MA, Al-Shehbaz IA, Franzke A, Mummenhoff K, Stamatakis A, Koch MA. 2014 BrassiBase: introduction to a novel knowledge database on Brassicaceae evolution. Plant Cell Physiol 55: e3. 24259684 10.1093/pcp/pct158

[JIAOGR213652C9] Koch MA, Kiefer M, German DA, Al-Shehbaz IA, Franzke A, Mummenhoff K, Schmickl R. 2012 BrassiBase: tools and biological resources to study characters and traits in the Brassicaceae—version 1.1. Taxon 61: 1001–1009.

[JIAOGR213652C10] Koren S, Phillippy AM. 2015 One chromosome, one contig: complete microbial genomes from long-read sequencing and assembly. Curr Opin Microbiol 23: 110–120. 25461581 10.1016/j.mib.2014.11.014

[JIAOGR213652C11] Lam ET, Hastie A, Lin C, Ehrlich D, Das SK, Austin MD, Deshpande P, Cao H, Nagarajan N, Xiao M, 2012 Genome mapping on nanochannel arrays for structural variation analysis and sequence assembly. Nat Biotechnol 30: 771–776. 22797562 10.1038/nbt.2303 PMC3817024

[JIAOGR213652C12] Li H, Durbin R. 2009 Fast and accurate short read alignment with Burrows-Wheeler transform. Bioinformatics 25: 1754–1760. 19451168 10.1093/bioinformatics/btp324 PMC2705234

[JIAOGR213652C13] Li H, Handsaker B, Wysoker A, Fennell T, Ruan J, Homer N, Marth G, Abecasis G, Durbin R, 1000 Genome Project Data Processing Subgroup. 2009 The Sequence Alignment/Map format and SAMtools. Bioinformatics 25: 2078–2079. 19505943 10.1093/bioinformatics/btp352 PMC2723002

[JIAOGR213652C14] Melters DP, Bradnam KR, Young HA, Telis N, May MR, Ruby JG, Sebra R, Peluso P, Eid J, Rank D, 2013 Comparative analysis of tandem repeats from hundreds of species reveals unique insights into centromere evolution. Genome Biol 14: R10. 23363705 10.1186/gb-2013-14-1-r10 PMC4053949

[JIAOGR213652C15] Nordström KJV, Albani MC, James GV, Gutjahr C, Hartwig B, Turck F, Paszkowski U, Coupland G, Schneeberger K. 2013 Mutation identification by direct comparison of whole-genome sequencing data from mutant and wild-type individuals using k-mers. Nat Biotechnol 31: 325–330. 23475072 10.1038/nbt.2515

[JIAOGR213652C16] Pendleton M, Sebra R, Pang AWC, Ummat A, Franzen O, Rausch T, Stütz AM, Stedman W, Anantharaman T, Hastie A, 2015 Assembly and diploid architecture of an individual human genome via single-molecule technologies. Nat Methods 12: 780–786. 26121404 10.1038/nmeth.3454 PMC4646949

[JIAOGR213652C17] Putnam NH, O'Connell BL, Stites JC, Rice BJ, Blanchette M, Calef R, Troll CJ, Fields A, Hartley PD, Sugnet CW, 2016 Chromosome-scale shotgun assembly using an in vitro method for long-range linkage. Genome Res 26: 342–350. 26848124 10.1101/gr.193474.115 PMC4772016

[JIAOGR213652C18] Quick J, Quinlan AR, Loman NJ. 2014 A reference bacterial genome dataset generated on the MinION™ portable single-molecule nanopore sequencer. GigaScience 3: 22. 25386338 10.1186/2047-217X-3-22 PMC4226419

[JIAOGR213652C19] Roach JC, Boysen C, Wang K, Hood L. 1995 Pairwise end sequencing: a unified approach to genomic mapping and sequencing. Genomics 26: 345–353. 7601461 10.1016/0888-7543(95)80219-c

[JIAOGR213652C20] Schranz ME, Lysak MA, Mitchell-Olds T. 2006 The ABC's of comparative genomics in the Brassicaceae: building blocks of crucifer genomes. Trends Plant Sci 11: 535–542. 17029932 10.1016/j.tplants.2006.09.002

[JIAOGR213652C21] Schwartz DC, Li X, Hernandez LI, Ramnarain SP, Huff EJ, Wang YK. 1993 Ordered restriction maps of *Saccharomyces cerevisiae* chromosomes constructed by optical mapping. Science 262: 110–114. 8211116 10.1126/science.8211116

[JIAOGR213652C22] Tang H, Lyons E, Town CD. 2015 Optical mapping in plant comparative genomics. GigaScience 4: 3. 25699175 10.1186/s13742-015-0044-y PMC4332928

[JIAOGR213652C23] VanBuren R, Bryant D, Edger PP, Tang H, Burgess D, Challabathula D, Spittle K, Hall R, Gu J, Lyons E, 2015 Single-molecule sequencing of the desiccation-tolerant grass *Oropetium thomaeum*. Nature 527: 508–511. 26560029 10.1038/nature15714

[JIAOGR213652C24] Willing E-M, Rawat V, Mandáková T, Maumus F, James GV, Nordström KJV, Becker C, Warthmann N, Chica C, Szarzynska B, 2015 Genome expansion of *Arabis alpina* linked with retrotransposition and reduced symmetric DNA methylation. Nat Plants 1: 14023. 27246759 10.1038/nplants.2014.23

