# Supplementary material for: Improving and correcting the contiguity of long-read genome assemblies of three plant species using optical mapping and chromosome conformation capture data
Source: Genome Res. 2017 May;27(5):778–86. doi: 10.1101/gr.213652.116 (PMC5411772; doi:10.1101/gr.213652.116)
Supplement: Supplemental Material [file supp_27_5_778__index.html]

Improving and correcting the contiguity of long-read genome assemblies of three plant species using optical mapping and chromosome conformation capture data — Improving and correcting the contiguity of long-read genome assemblies of three plant species using optical mapping and chromosome conformation capture data — Supplemental Material 

# Improving and correcting the contiguity of long-read genome assemblies of three plant species using optical mapping and chromosome conformation capture data

## Supplemental Material

- Supplemental\_Table\_S4.xlsx
- Supplemental\_Scripts.zip
- Supplemental\_Data.zip
- Supplemental\_Methods\_Figures.pdf
