# Supplementary material for: Improving and correcting the contiguity of long-read genome assemblies of three plant species using optical mapping and chromosome conformation capture data
Source: Genome Res. 2017 May;27(5):778–86. doi: 10.1101/gr.213652.116 (PMC5411772; doi:10.1101/gr.213652.116)
Supplement: Supplemental Material [file supp_gr.213652.116_Supplemental_Methods_Figures.pdf]

Supplemental Material for:

Improving and correcting the contiguity of long-read genome assemblies of three related plant species using optical mapping and chromatin conformation capture data

Jiao et al.

This pdf includes

Supplemental Material and Methods
Supplemental Figures S1 – S6
Supplemental Tables S1 – S3 and S5 – S13

Supplemental Material and Methods

Plant sample preparation

Plants from all three species were grown on standard soil under greenhouse conditions. The seeds were stratified on soil for two to three days at 4 °C straight after sowing.

For Illumina sequencing, young leaf material from single plants was collected after at least three weeks of plant growth and snap frozen in liquid nitrogen immediately after sampling. DNA was prepared with QIAGEN DNeasy plant mini kit according to the manufacturer’s instructions and quality checked on an agarose gel prior to library preparation.

For PacBio sequencing, optical mapping and chromatin capture multiple plants were grown on standard soil. When two to four weeks old plants were covered for two to three days. After dark treatment young leaf tissue was collected and snap frozen immediately in liquid nitrogen.

PacBio sequencing

Genomic DNA was isolated from 500 mg frozen leave tissue with the NucleoSpin Plant II Maxi kit (Macherey-Nagel, Düren, Germany) as recommended. Isolated high molecular weight DNA was quantified by fluorometry (Qubit, Thermo Fisher Scientific, Waltham, U.S.A.) and assessed for quality by 0.8% (w/v) agarose gel electrophoresis or with the genomic assay on the TapeStation (Agilent, Waldbronn, Germany). Single Molecule Real Time (SMRT) bell libraries were prepared according to the "20 kb Template Preparation Using BluePippin Size-Selection System" as recommended by Pacific Biosciences (Palo Alto, U.S.A). After damage-repair the libraries were size-selected on a BluePippin system (0.75% (w/v) agarose gel cassette, dye-free, S1 marker, high pass 6-10 kb vs3 protocol) to remove library fragments smaller than 10kb. Then libraries were recovered by PB AMPure beads, quantified by the high sensitivity fluorometric assay (Qubit, Thermo Fisher Scientific, Waltham, U.S.A.) and quality assessed by DNA12000 assay on a 2100 Bioanalyser (Agilent, Waldbronn, Germany). SMRT bell templates were bound to P6 polymerase using the DNA polymerase binding kit P6 v2 primers. Polymerase-template complexes were bound to magnetic beads using the Magbead Binding Kit and sequencing was carried out on the PacBio RS II sequencer using C4v1 sequencing reagents with movie lengths of 360 min on SMRT cells.

Illumina sequencing

Illumina paired-end library of *E. syriacum* was generated and sequenced on a HiSeq2500 instrument at the Max Planck-Genome centre, Cologne, Germany. Illumina reads of *A. alpina* and *C. planisiliqua* were taken from earlier studies (Willing et al. 2015; Bewick et al. 2016). The paired-end reads were used to estimated heterozygosity levels based on 25-mer frequencies calculated with Jellyfish (Marçais and Kingsford 2011) and genomescope.R (https://github.com/schatzlab/genomescope). Mate-pair libraries were constructed, quality controlled by alignment to the constructed contigs, and chosen for sequencing as described earlier (Heavens et al. 2015). Raw reads were pre-processed using a pipeline based on Nextclip (Leggett et al. 2014).

Genetic map generation

An F_2 mapping population containing 389 individuals was obtained from three self-pollinated F_1 hybrids between between two *A. alpina* accessions from the French Alps. DNA was extracted from each F_2 plant using the Qiagen DNeasy Plant Mini Kit according to manual. Libraries for genotyping-by-sequencing were prepared and barcoded ApeKI restriction fragments were sequenced at the Genomic Diversity Facility at Cornell University (Ithaca NY, USA) (Elshire et al. 2011).

Sequencing reads starting with the ApeKI recognition site were cleaned with Cutadapt (Martin 2011) and Trimmomatic (Bolger et al. 2014) and then mapped to the genome using BWA (Li and Durbin 2009). Consensus calling was performed with samtools and bcftools (Li et al. 2009) on positions with at least five reads and a quality of 25. Sites were considered as homozygous if the major allele had a frequency of at least 0.9 otherwise the site was labelled as heterozygous. All genotypes that did not match the parental alleles were considered as missing values. A genotype table, containing only markers that were homozygous in both parents, was filtered for individuals that had missing values at more than 80% of initial markers and for markers for which less than 70% of individuals were genotyped or did not have the expected segregation pattern according to a Chi-square test ($P <$ 0.0001). The resulting genotypes were used for linkage analysis using JoinMap v4.0. Markers were grouped using Maximum likelihood option at a minimum LOD score of 3.0 and maximum recombination fraction of 0.25 as general linkage criteria to establish linkage groups. Kosambi's function was applied to convert recombination percentages to centiMorgan map unit distances (Kosambi 1943).

Optical mapping

Earlham Institute's Platforms and Pipelines group followed IrysPrep™ Fix'n'Blend Plant DNA extraction protocol supplied by Bionano Genomics. 2.5 g of fresh young leaves were fixed with 2% formaldehyde. After washing, leaves are disrupted and homogenized in the presence of isolation buffer. The isolation buffer contains PVP10 and BME to prevent oxidation of polyphenols. Triton X-100 is added to facilitate the release of nuclei from the broken cells. The nuclei are then purified on a Percoll cushion. A nuclei phase is taken and washed several times in isolation buffer before embedding into low melting point agarose. Two plugs of 90 ul were cast using the Chef Mammalian Genomic DNA Plug Kit (Bio-Rad 170-3591). Once set at 4° the plugs were added to a lysis solution containing 200 µl proteinase K (QIAGEN 158920) and 2.5ml of Bionano lysis buffer in a 50 ml conical tube. These were put at 50°C for two hours on a thermomixer, making a fresh proteinase K solution to incubate overnight. The 50 ml tubes were then removed from the thermomixer for five minutes before 50 µl RNAse A (Qiagen158924) was added and the tubes returned to the thermomixer for a further hour at 37°C. The plugs were then washed seven times in Wash Buffer supplied in Chef kit and seven times in 1xTE. One plug was removed and melted for two minutes at 70°C followed by five minutes at 43°C before adding 10 ul of 0.2 U/µl of GELase (Cambio Ltd G31200). After 45 minutes at 43°C the melted plug was dialysed on a 0.1 uM membrane (Millipore VCWP04700) sitting on 15 ml of 1xTE in a small petri dish. After two hours the sample was removed with a wide bore tip and mixed gently five times and left overnight at 4°C. A small amount was removed to QC on an Opgen Argus Q-Card and Qubit HS for the DNA concentration. 300 ng of DNA was taken into the NLRS (Nick, Label, Repair and Stain) reaction using 1 µl Nt.BspQI (NEB R0644S). Following the NLRS reaction 16 ul was loaded onto a single flow cell on a Bionano chip. The Chip loading was optimised and run for 30 cycles on the Bionano Irys using ICS1.6. The same chip was run a total of five times. Images were converted to .bnx files using AutoDetect 2.1.0.6656 before analysis.

Estimation of centromeric regions

Using the cytogenetic maps and a whole-genome alignment (using nucmer from MUMmer version 3.23 (Kurtz et al. 2004) using "--mum -l 40 -g 90 -c 90 -b 200") of *A. lyrata* and *A. thaliana* (Schranz et al. 2006) we defined the centromere positions of *A. lyrata* following the centromere positons of *A. thaliana* (Arabidopsis Genome Initiative 2000). The adjacent alignment block of each of the genome alignments of *A. lyrata*

and the three assemblies (again using nucmer parameters "--mum -l 20 -g 90 -c 65 -b 200") were chained and extended into syntenic blocks. Scaffolds with syntenic blocks corresponding to both flanking sides of a centromere in *A. lyrata* were considered as centromere spanning. While those scaffolds with syntenic blocks corresponding to only one side of *A. lyrata* centromere were considered as partial centromeres. In addition we checked whether the assemblies contained centromeric tandem repeats (Melters et al. 2013), For this we performed de novo prediction of tandem repeat arrays using Tandem Repeat Finder with parameter setting "1 1 2 80 5 200 2000 -h". The longest tandem arrays were selected and clustered to find the most abundant repeat units, which were defined as the candidate centromeric repeat.

Annotating and finalizing the assemblies

To allow general usage of these assembly resources, we further improved the assembly of *A. alpina* by splitting the remaining erroneous contigs and integrating comparative BAC hybridization data as described earlier (Willing et al. 2015) and annotated genes across all three genomes. For gene annotations of *E. syriacum* and *C. planisiliqua* we aligned the protein coding sequences of eight different Brassicaceae species (*A. thaliana, A. lyrata, Capsella rubella, Brassica rapa, Eutrema salsugineum, Schrenkiella parvula, A. alpina, Arabis montbretiana*) (Arabidopsis Genome Initiative 2000; Hu et al. 2011; Slotte et al. 2013; The Brassica rapa Genome Sequencing Project Consortium et al. 2011; Yang et al. 2013; Dassanayake et al. 2011; Willing et al. 2015) to the genomic scaffolds using Scipio (v1.4) (Keller et al. 2008) to obtain homology based gene models. In addition, we used three *ab initio* prediction methods for *de novo* gene finding including GlimmerHMM (v3.0) (Majoros et al. 2004), SNAP (v2013) (Korf 2004) and Augustus (v3.0) (Stanke and Waack 2003). The resulting annotations were combined into weighted consensus gene structures using EVidenceModeler (EVM) software (v2012) (Haas et al. 2008). Gene models of an earlier version of the *A. alpina* assembly were used to annotate genes in the new assembly. Genomic scaffolds were annotated for transposable elements using RepeatMasker (v4.0) and a custom Brassicaceae repeat library. Predicted consensus models were removed from the annotation, if their predicted coding sequence overlapped with an annotated TE.

References

Arabidopsis Genome Initiative. 2000. Analysis of the genome sequence of the flowering plant Arabidopsis thaliana. *Nature* **408**: 796–815.

Bewick AJ, Ji L, Niederhuth CE, Willing E-M, Hofmeister BT, Shi X, Wang L, Lu Z, Rohr NA, Hartwig B, et al. 2016. On the origin and evolutionary consequences of gene body DNA methylation. *Proc Natl Acad Sci* **113**: 9111–9116.

Bolger AM, Lohse M, Usadel B. 2014. Trimmomatic: a flexible trimmer for Illumina sequence data. *Bioinformatics* **30**: 2114–2120.

Dassanayake M, Oh D-H, Haas JS, Hernandez A, Hong H, Ali S, Yun D-J, Bressan RA, Zhu J-K, Bohnert HJ, et al. 2011. The genome of the extremophile crucifer Thellungiella parvula. *Nat Genet* **43**: 913–918.

Elshire RJ, Glaubitz JC, Sun Q, Poland JA, Kawamoto K, Buckler ES, Mitchell SE. 2011. A Robust, Simple Genotyping-by-Sequencing (GBS) Approach for High Diversity Species. *PLOS ONE* **6**: e19379.

Haas BJ, Salzberg SL, Zhu W, Pertea M, Allen JE, Orvis J, White O, Buell CR, Wortman JR. 2008. Automated eukaryotic gene structure annotation using EVidenceModeler and the Program to Assemble Spliced Alignments. *Genome Biol* **9**: R7.

Heavens D, Accinelli GG, Clavijo B, Clark MD. 2015. A method to simultaneously construct up to 12 differently sized Illumina Nextera long mate pair libraries with reduced DNA input, time, and cost. *BioTechniques* **59**: 42–45.

Hu TT, Pattyn P, Bakker EG, Cao J, Cheng J-F, Clark RM, Fahlgren N, Fawcett JA, Grimwood J, Gundlach H, et al. 2011. The Arabidopsis lyrata genome sequence and the basis of rapid genome size change. *Nat Genet* **43**: 476–481.

Keller O, Odronitz F, Stanke M, Kollmar M, Waack S. 2008. Scipio: Using protein sequences to determine the precise exon/intron structures of genes and their orthologs in closely related species. *BMC Bioinformatics* **9**: 278.

Korf I. 2004. Gene finding in novel genomes. *BMC Bioinformatics* **5**: 59.

Kosambi DD. 1943. The Estimation of Map Distances from Recombination Values. *Ann Eugen* **12**: 172–175.

Kurtz S, Phillippy A, Delcher AL, Smoot M, Shumway M, Antonescu C, Salzberg SL. 2004. Versatile and open software for comparing large genomes. *Genome Biol* **5**: R12.

Leggett RM, Clavijo BJ, Clissold L, Clark MD, Caccamo M. 2014. NextClip: an analysis and read preparation tool for Nextera Long Mate Pair libraries. *Bioinforma Oxf Engl* **30**: 566–568.

Li H, Durbin R. 2009. Fast and accurate short read alignment with Burrows-Wheeler transform. *Bioinformatics* **25**: 1754–1760.

Li H, Handsaker B, Wysoker A, Fennell T, Ruan J, Homer N, Marth G, Abecasis G, Durbin R, 1000 Genome Project Data Processing Subgroup. 2009. The Sequence Alignment/Map format and SAMtools. *Bioinformatics* **25**: 2078–2079.

Majoros WH, Pertea M, Salzberg SL. 2004. TigrScan and GlimmerHMM: two open source ab initio eukaryotic gene-finders. *Bioinformatics* **20**: 2878–2879.

Marçais G, Kingsford C. 2011. A fast, lock-free approach for efficient parallel counting of occurrences of k-mers. *Bioinformatics* **27**: 764–770.

Martin M. 2011. Cutadapt removes adapter sequences from high-throughput sequencing reads. *EMBnet.journal* **17**: 10–12.

Melters DP, Bradnam KR, Young HA, Telis N, May MR, Ruby JG, Sebra R, Peluso P, Eid J, Rank D, et al. 2013. Comparative analysis of tandem repeats from hundreds of species reveals unique insights into centromere evolution. *Genome Biol* **14**: R10.

Schranz ME, Lysak MA, Mitchell-Olds T. 2006. The ABC's of comparative genomics in the Brassicaceae: building blocks of crucifer genomes. *Trends Plant Sci* **11**: 535–542.

Slotte T, Hazzouri KM, Ågren JA, Koenig D, Maumus F, Guo Y-L, Steige K, Platts AE, Escobar JS, Newman LK, et al. 2013. The Capsella rubella genome and the genomic consequences of rapid mating system evolution. *Nat Genet* **45**: 831–835.

Stanke M, Waack S. 2003. Gene prediction with a hidden Markov model and a new intron submodel. *Bioinformatics* **19**: ii215-ii225.

The Brassica rapa Genome Sequencing Project Consortium, Wang X, Wang H, Wang J, Sun R, Wu J, Liu S, Bai Y, Mun J-H, Bancroft I, et al. 2011. The genome of the mesopolyploid crop species Brassica rapa. *Nat Genet* **43**: 1035–1039.

Willing E-M, Rawat V, Mandáková T, Maumus F, James GV, Nordström KJV, Becker C, Warthmann N, Chica C, Szarzynska B, et al. 2015. Genome expansion of Arabis alpina linked with retrotransposition and reduced symmetric DNA methylation. *Nat Plants* **1**: 14023.

Yang R, Jarvis DE, Chen H, Beilstein MA, Grimwood J, Jenkins J, Shu S, Prochnik S, Xin M, Ma C, et al. 2013. The Reference Genome of the Halophytic Plant Eutrema salsugineum. *Front Plant Sci* **4**: 46.

Supplemental figures

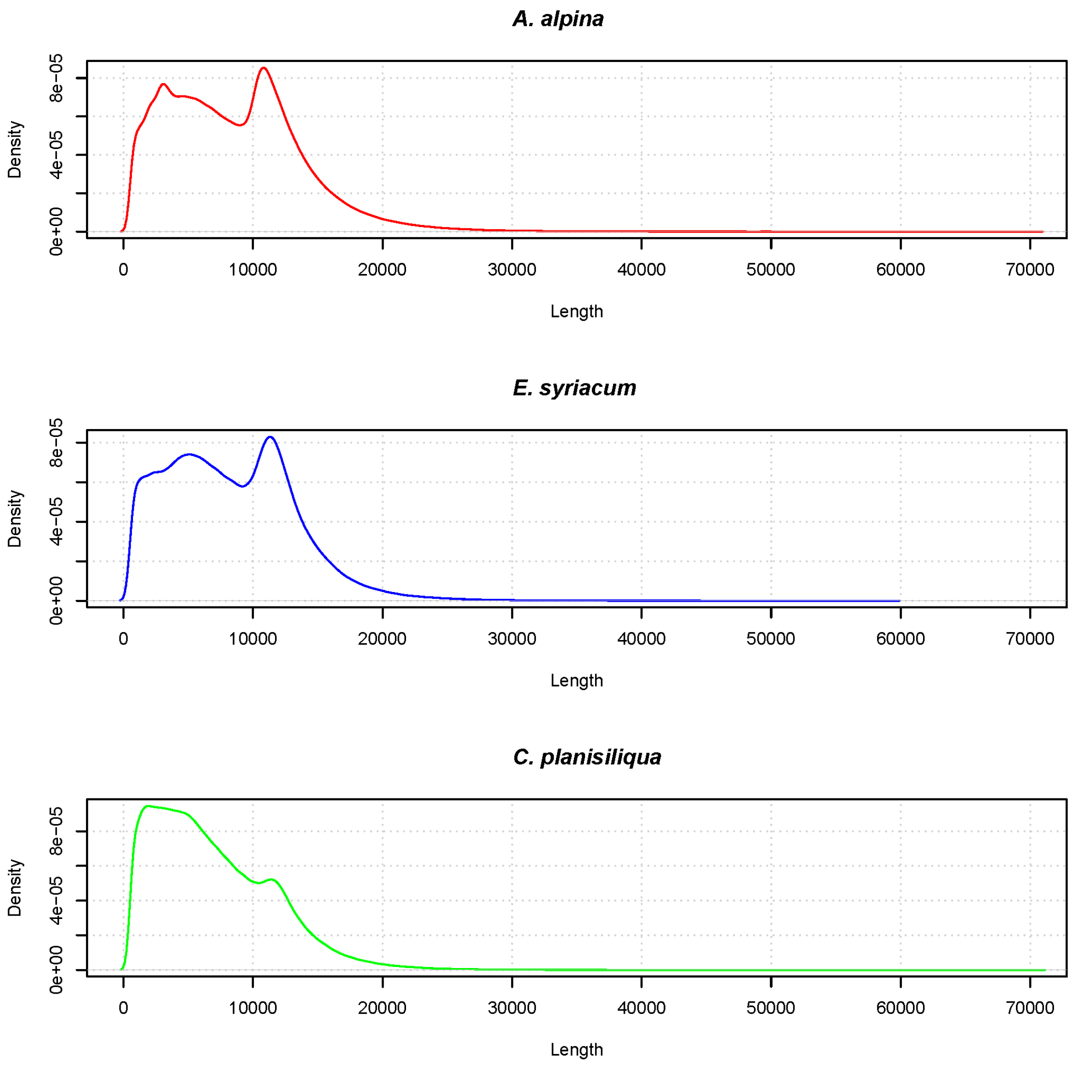

Figure S1. Length distribution of PacBio filtered subreads for the three genomes.

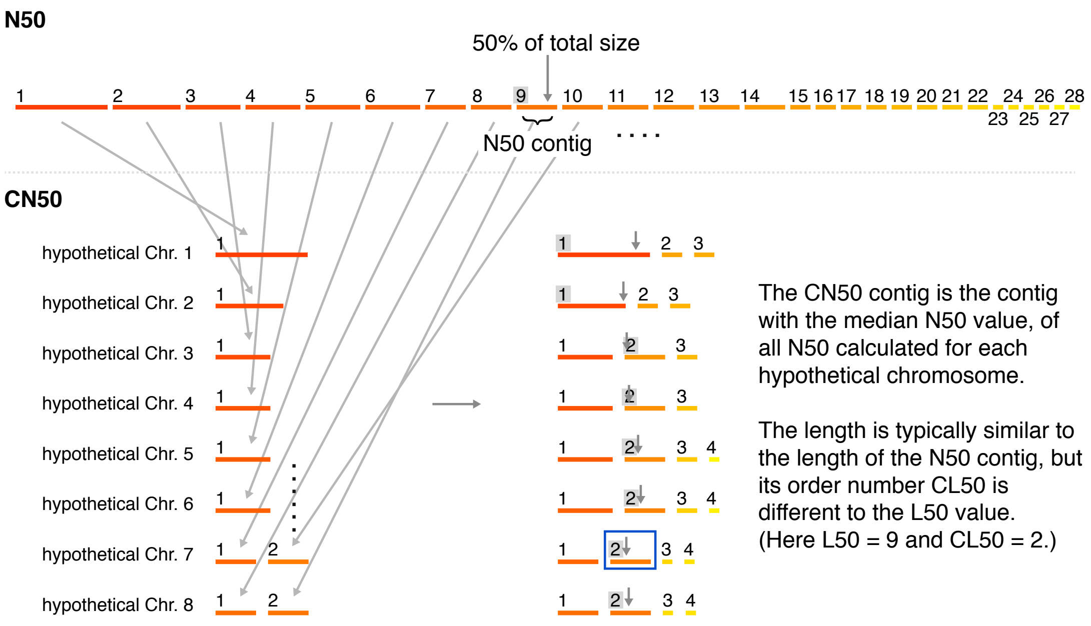

Figure S2. Definition of CN50 and CL50 statistics. N50 and L50 refer to one particular contig of a sequence assembly. This contig along with all longer contigs make up more than 50% of the total assembly size. The length of this contig is given by the N50 value, whereas its order number (in an ordering where contigs are sorted by length) is given as L50 (though some people prefer to annotate it vice versa). However, even in perfect assemblies, the L50 is not 1 (as it optimally would be), as the number of chromosomes limits the L50 value. This effect is marginal if an assembly consists of many contigs, however, in assemblies with high contiguity, this effect can be drastic and interfere with the interpretation of the L50 value.

CN50 and CL50 normalize the N50 statistics for chromosome number (n). For this, the contigs are sorted to hypothetical chromosomes, where the first chromosome is assigned the longest contig, the second chromosome the second longest and so on. The n+1 longest contig is then assigned to the n-th chromosome again (in the above example contig #9 is assigned to hypothetical chromosome 8) and the n+2 longest contig is assigned to chromosome n-2 (here contig #10 is assigned to chromosome 7), until no more contigs are left. For each of the n contig sets N50 is calculated and the median of these values describes the CN50 value. The order number (L50) of the respective CN50 contig (shown in the blue box) in the hypothetical chromosome finally describes the CL50 value.

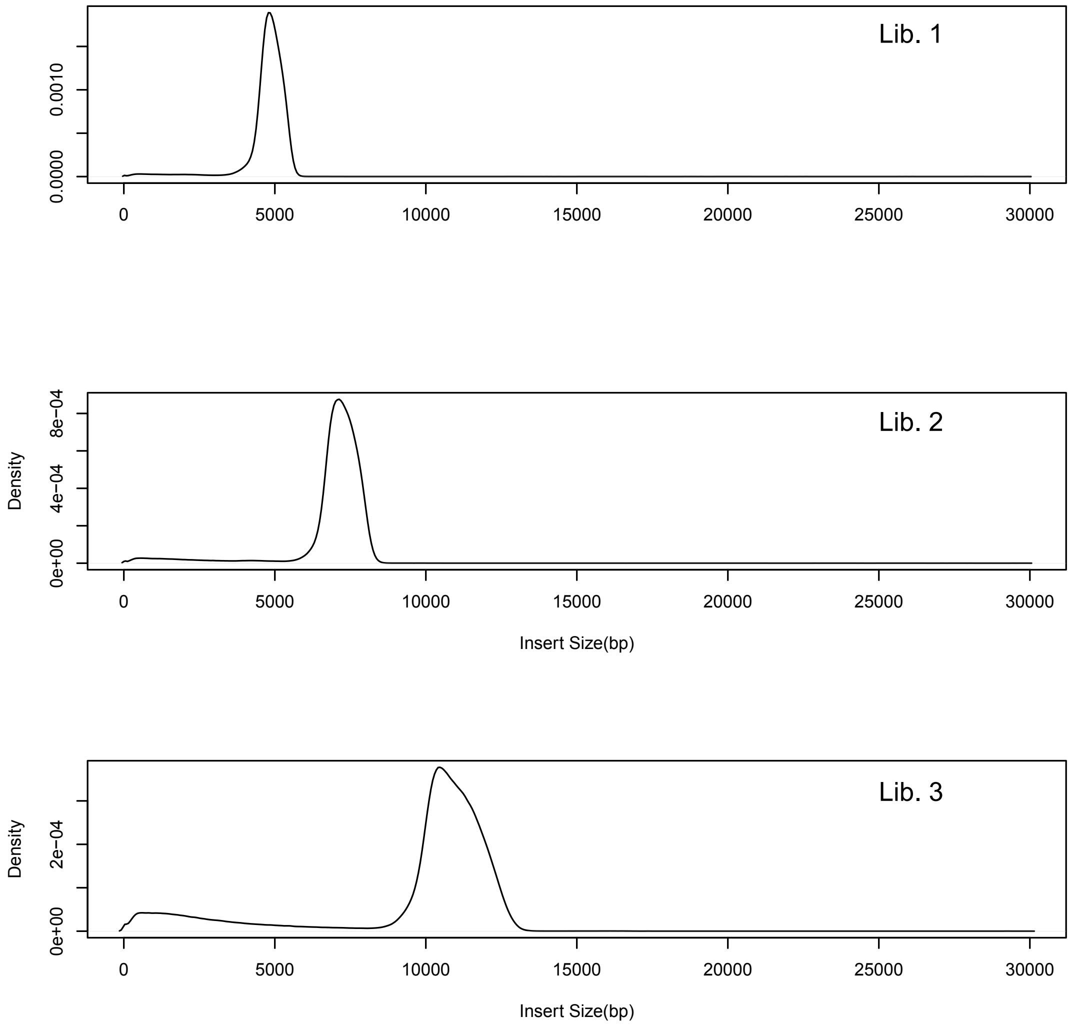

Figure S3. Insert size density distribution of the three mate-pair libraries generated for *A. alpina*.

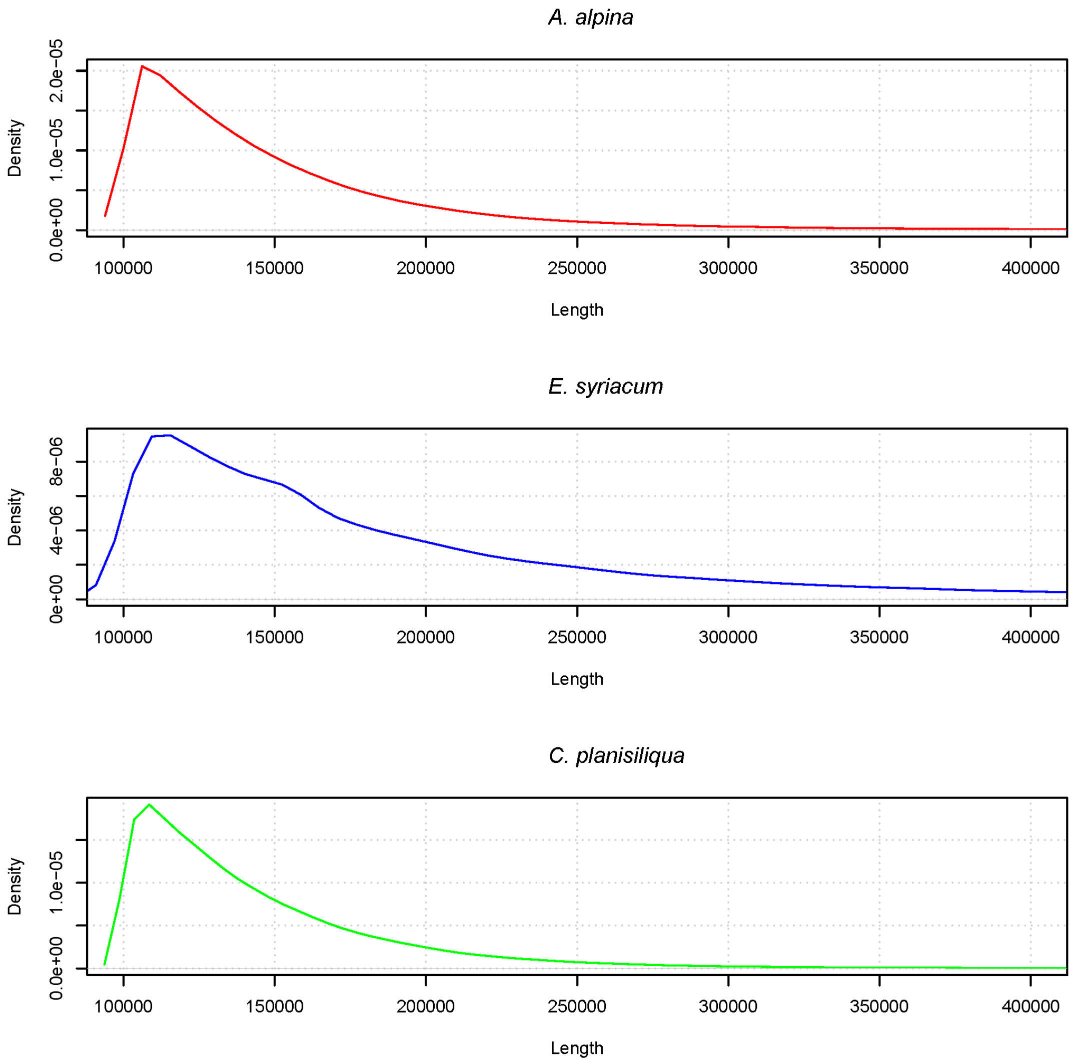

Figure S4. Length distribution of optical mapping molecules for the three genomes.

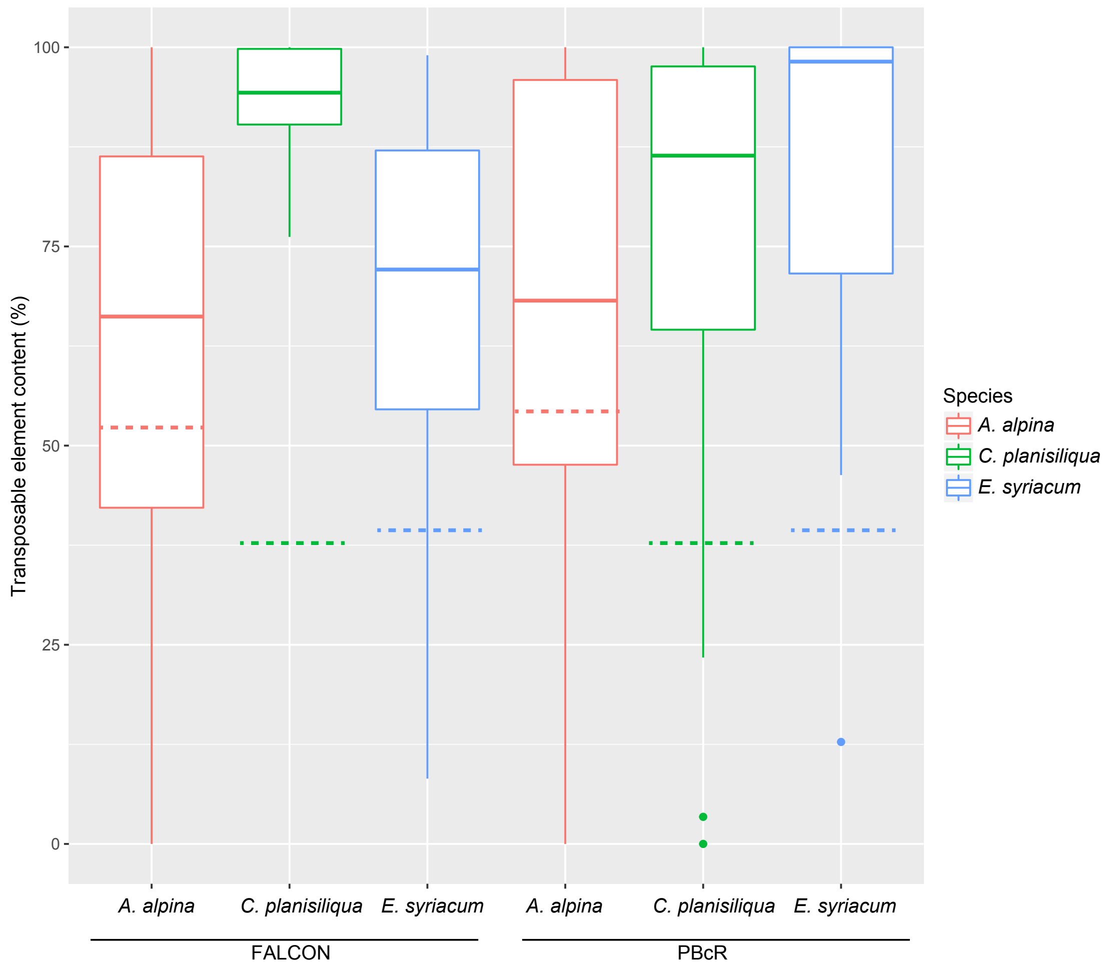

Figure S5. Distribution of transposable element content (%) in misassebled regions in the six initial assemblies (three species, two assembly tools) as compared to the average TE content density across the assembly (indicated with dashed lines).

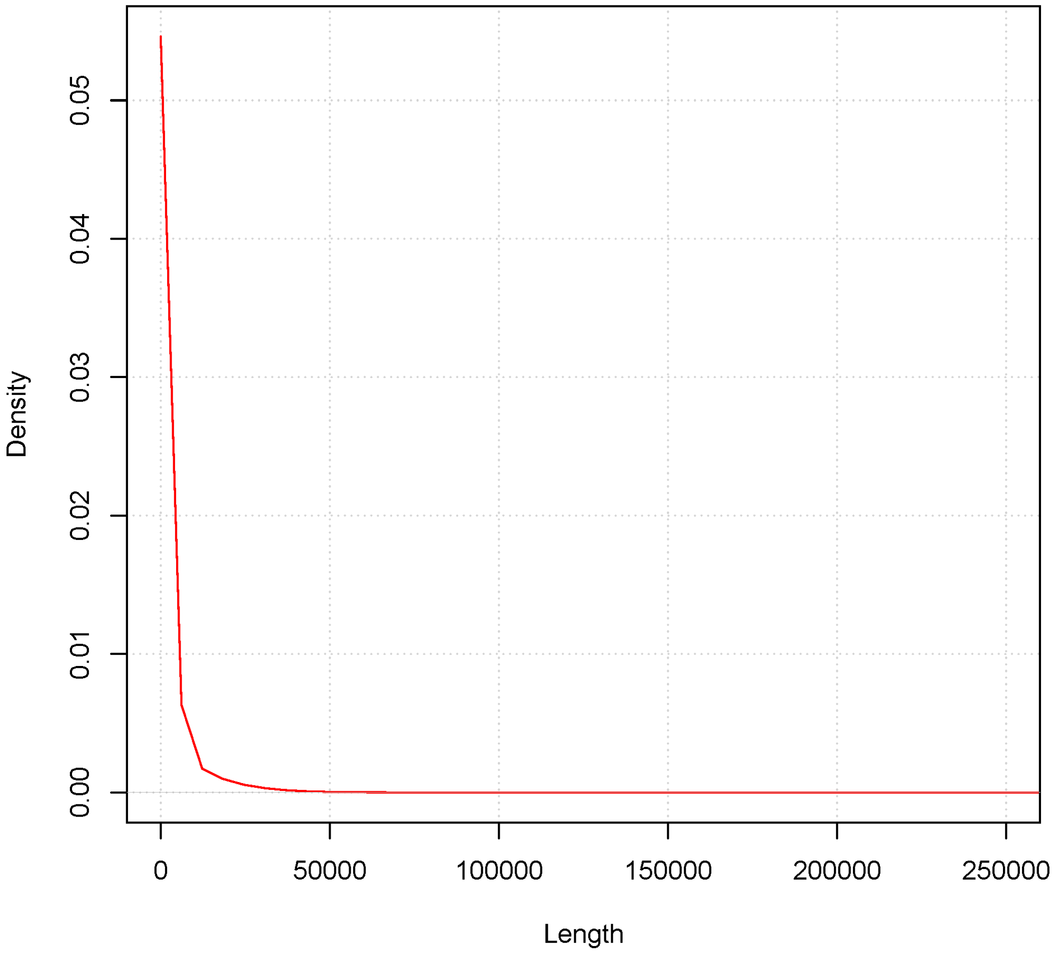

Figure S6. Insert size distribution of the Dovetail Genomics data of *A. alpina*.

Supplemental tables

Table S1. PacBio raw polymerase reads and filtered subreads statistics.

	A. alpina		*E. syriacum*		*C. planisiliqua*	
	raw reads	subreads	raw reads	subreads	raw reads	subreads
SMRT Cells	35	35	30	30	18	18
Total bases (Gb)	38.4	32.1	14.7	12.3	12.7	12
Total number (M)	5.3	3.8	4.5	1.8	2.7	1.5
Length N50 (kb)	18.7	11.3	10.4	10.8	14.5	11.1
Length mean (kb)	7.3	8.5	3.3	6.9	4.7	7.9
Coverage	102.3	85.5	55.6	46.6	56.6	53.5

Table S2. PacBio assembly nucleotide-level accuracy estimation.

	A. alpina		*E. syriacum*		*C. planisiliqua*	
	Falcon	PBcR	Falcon	PBcR	Falcon	PBcR
Mismatch	580	468	275	603	1,429	624
Indel	3,479	2,312	9,274	9,631	9,945	4,640
Error rate	0.0012%	0.0008%	0.0042%	0.0045%	0.0065%	0.0031%

Table S3. Mate-pair library read statistics.

	reads	mapped reads		mapped pairs		inter-contig pairs	
		Falcon	PBcR	Falcon	PBcR	Falcon	PBcR
Lib. 1 (5 kb)	50,804,106	91.7%	94.2%	86.3%	89.7%	19.6%	23.9%
Lib. 2 (7 kb)	50,138,688	90.3%	92.7%	83.6%	86.9%	20.4%	24.3%
Lib. 3 (10kb)	26,492,772	87.0%	89.5%	77.3%	81.0%	20.5%	24.5%

Inter-contig pairs: read pairs mapped on different contigs

Table S4. Marker sequences of the *A. alpina* mapping population.

Shown in additional file.

Table S5. Optical mapping data and consensus map statistics.

	Number of maps	Avg. map length(kb)	Coverage	Assembly size(Mb)	N50(kb)	L50	Nick sites / 100kb
A. alpina	1,729,537	157	722	322.8	624.6	166	9.6
E. syriacum	810,303	145	446	233.8	924.3	77	11.2
C. planisiliqua	461,383	200	410	199.7	1,474.2	41	12.3

Table S6. Consensus map (c-map) alignment statistics.

	A. alpina		*E. syriacum*		*C. planisiliqua*	
	Falcon	PBcR	Falcon	PBcR	Falcon	PBcR
Aligned c-map number	601	604	318	315	156	152
Aligned c-map length (%)	97.3	97.7	97.5	97.1	90.1	89.2
Covered c-map length (%)	85.0	87.6	94.0	89.3	82.3	77.3
Aligned contig number	495	446	140	430	151	262
Aligned contig length (%)	91.2	87.2	98.8	93.8	92.8	89.8
Covered contig length (%)	77.9	75.8	94.1	87.5	89.7	85.7

Aligned c-map/contig length: the total length of consensus maps/contigs, which can be aligned by contigs/consensus maps. Covered c-maps/contigs length: the total length of consensus map/contig regions, which were covered by contigs/consensus maps.

Table S7. Misassembled regions are enriched for transposable elements (TEs). Misassemblies include all conflicting regions between optical mapping data and sequence contigs. TE-rich column describes how many of the misassembled regions harbor more TEs than the genome average.

Species	Assembler	Misassemblies	TE-rich
A. alpina	Falcon	63	43 (68%)
A. alpina	PBcR	47	36 (77%)
C. planisiliqua	Falcon	15	15 (100%)
C. planisiliqua	PBcR	23	20 (87%)
E. syriacum	Falcon	7	6 (86%)
E. syriacum	PBcR	35	34 (97%)
**Sum**	**Falcon**	**85**	**64 (75%)**
**Sum**	**PBcR**	**105**	**90 (86%)**

Table S8. Location of rDNA and centromeric repeat arrays.

species	scaffold	scaffold length	array start	array end	unit number	unit length	type
A. alpina	scaffold_113	318,510	3,526	69,721	114	119	5S
A. alpina	scaffold_397	24,131	45	23,774	49	119	5S
A. alpina	scaffold_443	21,612	206	21,553	42	119	5S
A. alpina	scaffold_648	15,052	168	14,847	31	119	5S
A. alpina	scaffold_364	26,159	395	25,761	22	119	5S
A. alpina	scaffold_358	26,695	338	26,560	19	119	5S
A. alpina	scaffold_838	9,551	495	9,402	19	119	5S
A. alpina	scaffold_867	8,656	373	8,463	18	119	5S
A. alpina	scaffold_935	7,005	139	6,807	14	119	5S
A. alpina	scaffold_958	6,069	31	5,611	12	119	5S
A. alpina	scaffold_986	5,253	172	5,200	11	119	5S
A. alpina	scaffold_1023	4,275	29	4,066	9	119	5S
A. alpina	scaffold_15_1	1,417,828	1,960	56,842	6	5,350	NOR
A. alpina	scaffold_310	31,895	5,871	19,752	2.3	5,350	NOR
A. alpina	scaffold_474	20,252	2,662	19,196	2	5,350	NOR
A. alpina	scaffold_740	12,257	2,269	12,244	2	5,350	NOR
C. planisiliqua	scaffold_309	16,420	230	16,255	32	119	5S
C. planisiliqua	scaffold_18	3,088,089	3,078,359	3,087,683	19	119	5S
C. planisiliqua	scaffold_55	94,367	7,779	93,989	10	5,353	NOR

C. planisiliqua	scaffold_107	45,594	1,302	44,719	5.7	5,353	NOR
C. planisiliqua	scaffold_186	26,997	746	26,982	3.3	5,353	NOR
C. planisiliqua	scaffold_276	18,011	213	17,658	2.7	5,353	NOR
C. planisiliqua	scaffold_319	15,920	1,422	15,260	2	5,353	NOR
E. syriacum	scaffold_89	15,746	25	15,694	34	119	5S
E. syriacum	scaffold_92	15,505	170	15,132	32	119	5S
E. syriacum	scaffold_10	9,524,166	4,937,560	4,998,160	7.3	5,352	NOR
A. alpina	scaffold_5	8,313,247	8,311,056	8,313,247	4.4	495	CENT
A. alpina	scaffold_9	7,192,857	7,119,231	7,121,434	2.2	992	CENT
A. alpina	scaffold_9	7,192,857	7,165,320	7,192,857	55.3	509	CENT
A. alpina	scaffold_9	7,192,857	7,172,465	7,192,857	41.3	495	CENT
A. alpina	scaffold_38	3,081,905	3,019,109	3,020,774	3.4	495	CENT
A. alpina	scaffold_45	2,631,477	1	11,579	11.7	992	CENT
A. alpina	scaffold_56	1,882,626	1	1,253	2.5	495	CENT
A. alpina	scaffold_56	1,882,626	17,725	19,905	4.4	496	CENT
A. alpina	scaffold_56	1,882,626	45,692	48,362	2.7	990	CENT
A. alpina	scaffold_76	977,799	958,286	960,453	4.4	496	CENT
A. alpina	scaffold_91	632,066	26,864	31,481	4.7	990	CENT
A. alpina	scaffold_91	632,066	67,829	69,937	2.1	992	CENT
A. alpina	scaffold_91	632,066	89,451	91,919	4.9	496	CENT
A. alpina	scaffold_91	632,066	137,083	139,689	2.6	990	CENT
A. alpina	scaffold_91	632,066	148,864	149,954	2.2	494	CENT

A. alpina	scaffold_91	632,066	198,453	200,567	4.3	496	CENT
A. alpina	scaffold_91	632,066	206,431	209,096	5.4	496	CENT
A. alpina	scaffold_91	632,066	214,409	217,369	6.0	496	CENT
A. alpina	scaffold_91	632,066	228,424	229,525	2.2	495	CENT
A. alpina	scaffold_91	632,066	275,256	276,293	2.1	495	CENT
A. alpina	scaffold_91	632,066	300,707	301,921	2.5	495	CENT
A. alpina	scaffold_91	632,066	317,899	320,453	2.6	992	CENT
A. alpina	scaffold_91	632,066	325,857	327,338	3.0	495	CENT
A. alpina	scaffold_91	632,066	350,460	352,447	4.0	496	CENT
A. alpina	scaffold_91	632,066	369,166	371,648	5.0	496	CENT
A. alpina	scaffold_91	632,066	400,514	407,036	6.6	992	CENT
A. alpina	scaffold_91	632,066	441,588	448,065	13.1	496	CENT
A. alpina	scaffold_91	632,066	487,579	490,436	5.8	495	CENT
A. alpina	scaffold_91	632,066	527,132	529,024	3.8	496	CENT
A. alpina	scaffold_91	632,066	540,161	541,320	2.3	495	CENT
A. alpina	scaffold_91	632,066	552,453	553,501	2.1	495	CENT
A. alpina	scaffold_91	632,066	564,545	565,927	2.8	496	CENT
A. alpina	scaffold_91	632,066	604,422	608,152	3.8	990	CENT
A. alpina	scaffold_95	568,052	547,850	550,320	5.0	496	CENT
A. alpina	scaffold_95	568,052	556,005	557,812	3.6	496	CENT
A. alpina	scaffold_95	568,052	558,740	566,921	16.6	494	CENT
A. alpina	scaffold_104	448,397	345,740	346,823	2.2	494	CENT

A. alpina	scaffold_104	448,397	372,076	374,246	4.4	497	CENT
A. alpina	scaffold_104	448,397	399,500	400,687	2.4	497	CENT
A. alpina	scaffold_104	448,397	442,174	443,663	3.0	493	CENT
A. alpina	scaffold_109	359,092	420	3,045	5.3	493	CENT
A. alpina	scaffold_109	359,092	8,217	13,055	4.9	989	CENT
C. planisiliqua	scaffold_1	15,208,799	10,089,605	10,090,827	5.5	221	CENT
C. planisiliqua	scaffold_1	15,208,799	10,113,773	10,116,480	2.6	1,059	CENT
C. planisiliqua	scaffold_1	15,208,799	10,182,906	10,183,676	3.5	221	CENT
C. planisiliqua	scaffold_1	15,208,799	10,202,604	10,204,237	3.7	441	CENT
C. planisiliqua	scaffold_1	15,208,799	10,215,506	10,218,151	6.0	442	CENT
C. planisiliqua	scaffold_1	15,208,799	10,234,820	10,236,456	7.4	221	CENT
C. planisiliqua	scaffold_1	15,208,799	10,813,225	10,814,534	6.0	221	CENT
C. planisiliqua	scaffold_1	15,208,799	15,178,432	15,182,366	17.8	221	CENT
C. planisiliqua	scaffold_3	12,074,320	12,072,687	12,074,320	7.4	221	CENT
C. planisiliqua	scaffold_4	11,819,561	10,876,803	10,879,849	6.9	442	CENT
C. planisiliqua	scaffold_4	11,819,561	10,898,921	10,901,008	9.5	221	CENT
C. planisiliqua	scaffold_4	11,819,561	11,786,835	11,788,801	8.9	221	CENT
C. planisiliqua	scaffold_4	11,819,561	11,795,725	11,798,056	3.5	663	CENT
C. planisiliqua	scaffold_4	11,819,561	11,816,473	11,817,406	4.2	221	CENT
C. planisiliqua	scaffold_5	11,279,646	10,044,908	10,048,569	16.6	221	CENT
C. planisiliqua	scaffold_6	10,977,244	6,616,706	6,618,339	3.7	444	CENT
C. planisiliqua	scaffold_6	10,977,244	6,637,799	6,652,393	65.8	223	CENT

C. planisiliqua	scaffold_6	10,977,244	6,662,541	6,665,249	12.3	221	CENT
C. planisiliqua	scaffold_6	10,977,244	7,771,294	7,772,850	2.3	665	CENT
C. planisiliqua	scaffold_6	10,977,244	10,951,673	10,977,244	116.4	221	CENT
C. planisiliqua	scaffold_12	6,996,948	6,985,637	6,989,964	19.5	221	CENT
C. planisiliqua	scaffold_12	6,996,948	6,994,638	6,996,948	3.5	662	CENT
C. planisiliqua	scaffold_14	5,442,335	22,323	25,214	13.1	221	CENT
C. planisiliqua	scaffold_14	5,442,335	50,727	51,662	2.1	441	CENT
C. planisiliqua	scaffold_14	5,442,335	3,457,147	3,458,221	4.9	220	CENT
C. planisiliqua	scaffold_17	3,665,691	3,662,919	3,665,691	4.2	663	CENT
C. planisiliqua	scaffold_18	3,088,089	545	2,783	5.1	439	CENT
C. planisiliqua	scaffold_18	3,088,089	3,127	7,527	20.1	220	CENT
C. planisiliqua	scaffold_18	3,088,089	11,140	12,990	8.4	221	CENT
C. planisiliqua	scaffold_21	1,970,407	1	25,718	38.9	662	CENT
C. planisiliqua	scaffold_21	1,970,407	125,391	143,691	10.4	1,765	CENT
C. planisiliqua	scaffold_21	1,970,407	290,182	291,688	3.4	442	CENT
C. planisiliqua	scaffold_21	1,970,407	317,023	317,943	4.2	221	CENT
C. planisiliqua	scaffold_21	1,970,407	499,090	499,748	3.0	220	CENT
C. planisiliqua	scaffold_23	1,380,778	2	1,418	6.4	221	CENT
C. planisiliqua	scaffold_23	1,380,778	7,537	35,224	41.7	659	CENT
C. planisiliqua	scaffold_23	1,380,778	276,925	280,180	3.7	882	CENT
C. planisiliqua	scaffold_23	1,380,778	292,068	294,546	5.7	442	CENT
C. planisiliqua	scaffold_37	230,006	21	1,790	2.7	656	CENT

C. planisiliqua	scaffold_37	230,006	13,495	18,680	23.5	221	CENT
C. planisiliqua	scaffold_37	230,006	131,068	143,114	54.6	221	CENT
C. planisiliqua	scaffold_37	230,006	144,218	152,347	7.4	1,104	CENT

5S: 5S rDNA arrays.

NOR: nucleolus organizer region, including 18S, 5.8S and 25S rDNA, only those with at least two units were shown.

CENT: putative centromeric repeat arrays. Only scaffolds more than 200 kb were shown.

Table S9. Location of telomeric repeat arrays.

species	scaffold	scaffold length	array start	array end	unit number	unit sequence
A. alpina	scaffold_161	91,596	1	1,980	283.3	AAACCCT
A. alpina	scaffold_31	3,882,864	3,880,881	3,882,863	284.7	TAGGGTT
C. planisiliqua	scaffold_8	9,101,398	9,097,573	9,101,397	550	AGGGTTT
C. planisiliqua	scaffold_7	9,129,079	9,125,697	9,129,079	485.6	GTTTAGG
C. planisiliqua	scaffold_11	7,283,533	7,279,710	7,283,533	532.3	TAGGGTT
C. planisiliqua	scaffold_1	15,208,799	1	3,224	462.9	AAACCCT
C. planisiliqua	scaffold_2	12,270,481	1	2,816	402.3	AACCCTA
E. syriacum	scaffold_11	8,766,530	8,748,992	8,766,530	2509	TTTAGGG
E. syriacum	scaffold_12	6,520,592	6,510,080	6,520,592	1507.3	GTTTAGG
E. syriacum	scaffold_9	12,372,032	1	6,880	991.4	CCCTAAA
E. syriacum	scaffold_6	17,487,894	17,481,731	17,487,894	877.7	TTTAGGG
E. syriacum	scaffold_3	20,634,497	20,628,542	20,634,497	851.7	GGTTTAG
E. syriacum	scaffold_4	18,658,056	18,652,321	18,658,056	810.9	TTAGGGT
E. syriacum	scaffold_7	14,560,423	14,555,641	14,560,423	688.1	TTTAGGG
E. syriacum	scaffold_16	4,329,799	1	2,111	304.4	ACCCTAA
E. syriacum	scaffold_2	21,647,715	1	700	102.4	AAACCCT

Table S10. Summary of protein-coding gene annotations.

	A. alpina	*E. syriacum*	*C. planisiliqua*
Gene number	29,470	33,001	34,766
Total gene length	75,645,144	51,262,375	52,103,293
Gene region percent	23.2%	22.7%	29.4%
Coding region percent	10.5%	14.9%	19.2%

Table S11. Summary of transposable element annotations.

	A. alpina		*E. syriacum*		*C. planisiliqua*	
	number of elements	percentage of sequence	number of elements	percentage of sequence	number of elements	percentage of sequence
SINE	5,996	0.36%	1,731	0.15%	441	0.09%
LINE	13,889	3.63%	8,872	2.46%	4,632	1.51%
LTR	78,148	29.01%	43,042	20.16%	27,599	18.53%
DNA	52,824	6.50%	27,592	5.21%	16,103	4.42%
Unclassified	80,500	10.98%	49,449	9.73%	26,625	11.98%

Table S12. Number and percent of perfectly aligned genes against each intermediate assembly.

	PacBio raw	PacBio polished	Illumina corrected	1st OM scaffolded
A. alpina	13,512	29,294	29,420	29,423
	45.850%	99.403%	99.830%	99.841%
E. syriacum	11,982	32,035	33,000	33,000
	36.308%	97.073%	99.997%	99.997%
C. planisiliqua	13,809	33,956	34,765	34,765
	39.720%	97.670%	99.997%	99.997%

Table S13. Number of mismatches and alignment gaps of genes blasted against each intermediate assembly.

		PacBio raw		PacBio polished		Illumina corrected		1st OM scaffolded	
		Mismatch	Gaps	mismatch	gaps	mismatch	gaps	mismatch	gaps
A. a.	Genes	47,168	94,066	46	320	13	32	0	4
A. a.	Exons	15,296	31,286	5	60	0	22	0	1
E. s.	Genes	10,506	84,397	13	1,068	0	0	0	0
E. s.	Exons	6,391	45,581	11	486	0	0	0	0
C. p.	Genes	20,391	75,389	103	1,170	0	0	0	0
C. p.	Exons	14,841	43,875	85	732	0	0	0	0

A. a.: *A. alpina* E. s.: *E. syriacum*; C. p.: *C. planisiliqua*
